# Genomic Epidemiology of Methicillin-Resistant *Staphylococcus aureus* in a Neonatal Intensive Care Unit

**DOI:** 10.1371/journal.pone.0164397

**Published:** 2016-10-12

**Authors:** Taj Azarian, Nizar F. Maraqa, Robert L. Cook, Judith A. Johnson, Christine Bailey, Sarah Wheeler, David Nolan, Mobeen H. Rathore, J. Glenn Morris, Marco Salemi

**Affiliations:** 1 College of Public Health and Health Professions and College of Medicine, Department of Epidemiology, University of Florida, Gainesville, FL, United States of America; 2 Emerging Pathogens Institute, University of Florida, Gainesville, FL, United States of America; 3 Infectious Diseases and Immunology, Wolfson Children’s Hospital, Jacksonville, FL, United States of America; 4 University of Florida Center for HIV/AIDS Research, Education and Service, University of Florida, College of Medicine, Jacksonville, FL, United States of America; 5 Department of Pathology, Immunology and Laboratory Medicine, University of Florida, Gainesville, FL, United States of America; 6 Division of Infectious Diseases, Department of Medicine, College of Medicine, University of Florida, Gainesville, FL, United States of America; University of Mississippi Medical Center, UNITED STATES

## Abstract

Despite infection prevention efforts, neonatal intensive care unit (NICU) patients remain at risk of Methicillin-resistant *Staphylococcus aureus* (MRSA) infection. Modes of transmission for healthcare-associated (HA) and community-associated (CA) MRSA remain poorly understood and may vary by genotype, hindering the development of effective prevention and control strategies. From 2008–2010, all patients admitted to a level III NICU were screened for MRSA colonization, and all available isolates were *spa*-typed. *Spa*-type t008, the most prevalent CA- genotype in the United States, *spa*-type t045, a HA- related genotype, and a convenience sample of strains isolated from 2003–2011, underwent whole-genome sequencing and phylodynamic analysis. Patient risk factors were compared between colonized and noncolonized infants, and virulence and resistance genes compared between *spa*-type t008 and non-t008 strains. Epidemiological and genomic data were used to estimate MRSA importations and acquisitions through transmission reconstruction. MRSA colonization was identified in 9.1% (177/1940) of hospitalized infants and associated with low gestational age and birth weight. Among colonized infants, low gestational age was more common among those colonized with t008 strains. Our data suggest that approximately 70% of colonizations were the result of transmission events within the NICU, with the remainder likely to reflect importations of “outside” strains. While risk of transmission within the NICU was not affected by *spa*-type, patterns of acquisition and importation differed between t008 and t045 strains. Phylodynamic analysis showed the effective population size of spa-type t008 has been exponentially increasing in both community and hospital, with s*pa*-type t008 strains possessed virulence genes not found among t045 strains; t045 strains, in contrast, appeared to be of more recent origin, with a possible hospital source. Our data highlight the importance of both intra-NICU transmission and recurrent introductions in maintenance of MRSA colonization within the NICU environment, as well as *spa*-type-specific differences in epidemiology.

## Introduction

In pediatric populations, the risk of methicillin-resistant *Staphylococcus aureus* (MRSA) colonization and infection is greatest among infants hospitalized in the neonatal intensive care unit (NICU). MRSA colonization is a well-recognized risk factor leading to infection, and colonized infants are sources for subsequent transmission to other NICU patients [1–3]. Despite interventions, NICU outbreaks continue to occur, and substantive questions remain about management of MRSA in this setting [4]. Recent studies have sought to determine the relative contribution of repeated importations (i.e., sporadic cases) and intra-hospital transmission (i.e., endemic or acquired cases) to rates of *S*. *aureus* colonization and infection. Results have been variable, depending on pathogen (e.g., MRSA vs. MSSA), outcome (e.g., colonization vs. infection), patient population (e.g., adult vs. pediatric), and setting (e.g., ICU vs. non-ICU) studied [5,6].

Throughout the last decade, surveillance data have demonstrated a shift in prevalent MRSA genotypes within healthcare facilities, illustrating the evolving epidemiology of this pathogen. Particularly, the most common community-associated (CA-) MRSA genotype, identified as USA300 by pulsed-field gel electrophoresis (PFGE), predominantly t008 by *spa*-typing, and belonging to multi-locus sequence type (MLST) ST-8, has increased in prevalence among hospitalized patients, including those in the NICU [7–9]. Until recently, this shift has largely been characterized using molecular typing methods including PFGE, MLST, and *spa*-typing. However, there has been increasing interest in genomic epidemiology studies based on phylogenetic and population genetic (phylodynamic) analysis of MRSA whole-genome sequencing (WGS) data, with earlier data demonstrating substantial diversity within *S*. *aureus* populations previously thought to be clonal [10–12].

**Fig 1:**
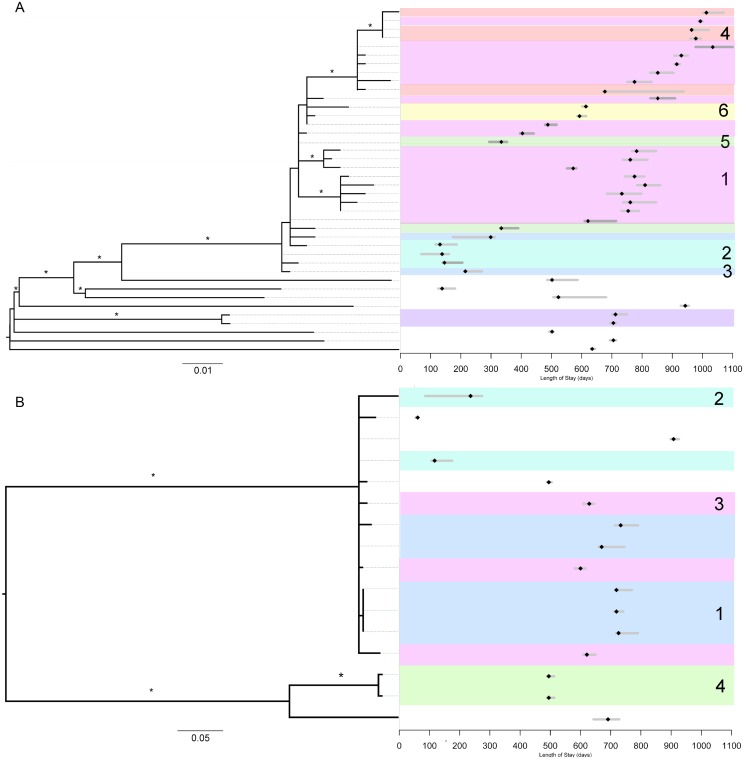
Maximum likelihood (ML) phylogenetic relationship among *spa*-type t045 and t008 MRSA isolates and patient length of stay with date of positive culture. The left side of the figure displays the ML phylogeny scaled in SNPs per site and ordered by decreasing genetic distance (i.e., closer phylogenetic relationships appear on top). Asterisks represent clades with bootstrap support values above 80% (i.e., well supported). The right side of the figure displays the corresponding clinical data for each patient. Bars associated with each tip (patient) of the phylogeny span the day of admission to day of discharge, and black point represents the collection date of positive surveillance culture. Shading and numbering of the epidemiological data corresponds to transmission clusters displayed is S7 Fig. A.) 40 *spa*-type t008 isolates obtained from infants hospitalized from 2008–2010 and corresponding length of stays. B.) 16 *spa*-type t045 isolates obtained from infants hospitalized from 2008–2010 and corresponding length of stays.

In the current study, we sought to explore the genomic epidemiology and population dynamics of MRSA strains circulating in a level III NICU and surrounding healthcare network, in an effort to determine the primary points of origin and risk factors for MRSA colonization [1,10,13,14]. To this extent, we assessed clinical, demographic, and bacterial genomic data obtained from multiple years of active MRSA surveillance (Fig 1), specifically focusing on the two most prevalent MRSA genotypes identified through *spa*-typing: *spa*-types t008 and t045, which represent historically defined CA- and HA-MRSA lineages respectively. We identified significant epidemiological and population genetic differences between *spa*-types t008 and t045. *Spa*-type t008 was the most prevalent among tested isolates, and phylogenetic analysis illustrated multiple introductions, with frequent intra-hospital transmission events. Carriage of *spa*-type t045 (ST-225), a seemingly rare *spa*-type genetically similar to prevalent HA-MRSA strains outside of the United States [15–17], appeared to have a more recent introduction and result from multiple introductions into the NICU. The results demonstrate variations in evolutionary history and transmission dynamics among prevalent genotypes of MRSA in the NICU and the continual colonization pressure from the hospital and surrounding community.

## Results and Discussion

### Clinical and Demographic Comparison of Infants

Since 2004, patients admitted to the 48 open-bed level III NICU in Florida (Hospital-A) underwent weekly MRSA screening of the nares until detection of colonization by the hospital’s clinical microbiology laboratory or discharge, using a standardized protocol [18]. We retrospectively assessed colonization and infection among NICU patients from January 1, 2008 through December 31, 2010. During the study period, 1940 infants were hospitalized in the NICU with an average length of stay (LOS) of 25.9 days (median = 14; range: 1–295) (S1A Fig). NICU occupancy remained constant with an average daily census of 45 patients. Surveillance identified 177 colonized patients, averaging 59 colonizations per year and translating to a colonization rate of 9.1% (Table 1). While monthly variations in colonization prevalence were observed, a median of six colonized patients was present daily, assuming that infants remained colonized until discharge even though decolonization was attempted (S2 Fig). The mean duration from admission to colonization was 12.0 days. Clinical and demographic covariates were compared between colonized and noncolonized infants hospitalized in the Hospital-A NICU (S1A Fig). Among the colonized infants, 96 (54.2%) were male and 137 (77.4%) were inborn (i.e. not transferred from another hospital). Infection was identified in 44 infants, including 33 with a prior colonization. There was an average of 7.5 days from colonization to infection. The 11 infants with infection and no prior colonization were excluded from the risk factor analysis since the temporality of colonization and infection could not be established. Utilizing multivariable logistic regression we identified several risk factors for MRSA colonization, consistent with previous reports [18,19]. Colonized infants had a significantly lower weight at birth, lower gestational age, and longer LOS compared to the noncolonized (Table 1). They were also more likely to be born by cesarean delivery and to be inborn. Higher birth weight and gestational age reduced odds of colonization, while caesarian birth and black race increased the odds (S1 Table). Of the 177 colonized infants, 100 (56.5%) isolates were available for *spa*-typing. The remaining isolates were either not viable or otherwise unavailable due to the retrospective study design. To assess the impact of missing data, we compared patient demographics and risk factors between patients with and without available isolates. Infants with isolates available for typing were not significantly different from those with unavailable isolates in terms of gender, race, birth weight, gestational age, LOS, and days to colonization (S2 Table). Colonization isolates were *spa*-type t008 (n = 54), t045 (n = 22), t002 (n = 7), and other (n = 17) (S3 Table and S3 Fig). The two most prevalent *spa*-types, t008 and t045, belong to clonal complex (CC) 8 and 5, respectively. In our study, we considered *spa*-type t008 isolates to belong to the prototypical CA-MRSA lineages, PFGE-type USA300, based on previous studies [9].

**Table 1:** Characteristics of cases (colonized infants) and controls (noncolonized infants).

	Cases (n = 177)	Controls (n = 1763)	Level of significance (p-value)*
Birth weight (median and range)	1.59 kg (0.46–4.38 kg)	2.42 kg (0.35–5.28 kg)	<0.001
Gestational age (median and range)	31 weeks (23–42 weeks)	35 (22–42 weeks)	<0.001
Length of stay (median and range)	49 days (1–295 days)	13 days (1–248 days)	<0.001
Birth by caesarean section	73.4% (130/177)	61.8% (1090/1763)	0.003
White race	57.6% (102/177)	69.7% (1229/1763)	0.004
Born off-site	22.6% (40/177)	33.0% (581/1763)	0.006
Multiple births	24.3% (43/177)	20.1% (355/1763)	0.23
Male gender	54.2% (96/177)	57.1% (1006/1763)	0.52
MRSA infection	18.6% (33/177)	n/a	
Days to positive MRSA (median and range)	12 (0–167)	n/a	

*As determined by Kruskal-Wallis and Chi2 tests

Univariate and multivariate logistic regression assessed risk factors for colonization between *spa*-type t008 and non-t008 colonized infants (S1B Fig). In univariate and multivariate models, infants colonized with *spa*-type t008 had a significantly lower gestational age, as compared with infants colonized with non-t008 isolates (Table 2 and S4 Table). In the final regression model, the gestational age, in turn, was a primary driver for increased LOS, so that infants who had t008 colonization had significantly longer LOS than infants colonized with other *spa*-types (S5 Table).

**Table 2:** Comparison of characteristics between patients with community genotype (*spa*-type *t008*) and healthcare genotypes (non-*spa*-type *t008*) ordered by statistical significance.

	*spa*-type *t008* (n = 54)	Non-*t008 spa*-types (n = 46)	Level of significance (p-value)*
Length of stay (median and range)	60 days (7–268)	44.5 days (6–182 days)	0.04
Gestational age (median and range)	28.5 weeks (23–42 weeks)	32 weeks (23–41 weeks)	0.08
Birth weight (median and range)	1.33 kg (0.54–3.64 kg)	1.7 kg (0.69–4.0 kg)	0.20
MRSA infection	20.4% (11/54)	10.9% (5/46)	0.31
White race	53.7% (29/54)	52.2% (24/46)	0.48
Multiple births	20.4% (11/54)	28.3% (13/46)	0.49
Birth by caesarean section	77.8% (42/54)	71.7% (33/46)	0.64
Days to positive MRSA (median and range)	15 (2–125)	13.5 (2–150)	0.72
Male gender	50.0% (27/54)	45.7% (21/46)	0.82
Born off-site	20.4% (11/54)	17.4% (8/46)	0.90

*As determined by Kruskal-Wallis and Chi2 tests

### Transmission Dynamics

Pathogen genomic data in the form of single-nucleotide polymorphisms (SNPs) may be used in combination with epidemiological data to infer transmission networks. However, the relationship between the temporality of cases and the evolutionary dynamics of a pathogen are complex [20]. For example, while inter-host diversity provides information about the heterogeneity of microbial strains circulating within a specific setting, delineating between importations (i.e., introduction of a cases into a setting) and acquisition (i.e., transmission events) may be difficult if transmission networks are partially observed or if the diversity between the source population has low diversity. Here, we aimed to distinguish importation from acquisition and understand the broader transmission dynamics of MRSA in the NICU by applying phylogenetic analysis and transmission tree reconstruction, specifically focusing on the two most prevalent genotypes, *spa*-types t008 and t045 (Fig 1C).

To assess hospital inter-host diversity we analyzed the WGS data of 40 t008 (81.5% of all available samples) and 16 t045 (72.7%) isolates (S1C Fig). Reference based alignments resulted in 345 SNPs for t008 and 209 for t045, with mean pairwise nucleotide differences of 32.0±3.6 and 51.7±1.4 SNPs, respectively. Both t045 and t008 datasets possess a bimodal distribution of pairwise SNP distances (S4 Fig). To further investigate the hospital and community level diversity of *spa*-type t008, we expanded the Hospital-A NICU sample to include 42 previously published of *spa*-type t008 genomes concurrently collected from five hospitals in the local healthcare network (Hospitals A, B, C, D, and E), as well as nine random pediatric intensive care (PICU) surveillance isolates from Hospital-A (Fig 1D and 1E and S6 Table) [10,21]. These isolates were previously collected to assess MRSA population structure among hospitals in the surrounding healthcare network [10] and serve as a sample of the community level diversity of *spa*-type t008. This dataset included 1929 SNPs with a mean pairwise nucleotide difference of 64.0±5.4. The distribution of pairwise SNP differences demonstrates a clear delineation in the bimodal distribution between Hospital-A NICU isolates and healthcare network (S5 Fig) and pairwise SNP distance and sampling site (NICU vs. healthcare network) are highly correlated (S6 Fig).

Putative importations of MRSA into the NICU and subsequent acquisition events were investigated through a Markov chain Monte Carlo (MCMC) method using genomic and epidemiological data to infer transmission trees [22]. Among colonized neonates, 9 [95% HPD: 6–12] and 8 [95% HPD: 8–10] importations were inferred for t008 and t045 strains, respectively (S7 Table). Additionally, 31 [95% HPD: 27–33] t008 and 8 [95% HPD: 6–8] t045 acquisitions were inferred. For t008 and t045 strains, the probability that a susceptible neonate becomes colonized given that a colonized patient is present in the NICU is comparable (3.94E-4 [95% HPD: 2.64E-4–5.49E-4] vs. 2.99E-4 [95% HPD: 1.07E-4–5.43E-4]), suggesting that *spa*-type alone does not affect a susceptible patient’s likelihood of being colonized. Interestingly, compared to clusters of t045 cases, the median nucleotide diversity between t008 transmission clusters was significantly lower (S7 Table). This finding is consistent with lower community level diversity of MRSA *spa*-type t008 serving as the source population for introductions into NICU.

We reconstructed transmission trees using the probability of importation and acquisition for each case (S7 Fig). The longest transmission chain among t008-colonized patients was comprised of 25 patients; however, we caution that in some cases, importation may have been plausible or missing isolates may have linked two or more individuals. As a result, the link between phylogenetically related but temporally distant cases potentially resulted from unsampled individuals or reservoirs, for example, a persistently or transiently colonized healthcare worker, as has previously been implicated [1,23]. Overall, while the colonization rate in the NICU population was consistent with previous studies [3], MCMC transmission reconstruction of all sequenced isolates suggested 69.6% of colonizations (~77.5% of t008 and ~50.0% of t045) were acquired.

The rooted ML phylogenies further illustrate the genetic relatedness among colonization isolates and the temporality of cases corresponding to the reconstructed transmission networks (Fig 1). Indeed, there are instances where pathogen genomic or epidemiological data alone would have suggested alternative transmission trees. Clearly, the use of a universal SNP cut-off to infer transmission events is seemingly inappropriate since the within and between transmission cluster diversity is not uniform among t045 and t008 populations, likely reflecting their respective population structures in the source reservoir (e.g., community or hospital). Finally, if we consider that unique *spa*-types (n = 12) represent independent importation events, combined with results from transmission reconstruction we estimate 29 separate importations of MRSA into the NICU. This reconstruction significantly differs from what would be inferred from epidemiological and *spa*-type data alone (S2 Fig), which imprecisely elucidates only four putative transmission chains among t008 colonized patients and misses entirely the two separate t045 populations in the NICU.

These results juxtapose with recent studies by Price *et al*. and by Long *et al*. that found colonization and infection, respectively, were not the result of recent intra-hospital transmission events [5,6]. Differences in patient populations and study setting likely explain contrasting results, considering a large proportion of infections in the study by Long *et al*. were present on admission, and only 17% were ICU patients. It has previously been suggested that CA- strains (e.g., PFGE type USA300 or *spa*-type t008) are more often acquired at birth, while HA- strains were acquired nosocomially [19].

In the current study, we observe clear differences in the transmission dynamics of two MRSA genotypes, which differed in population diversity, time of introduction into the healthcare community, and the proportion of cases resulting from importations and acquisitions. Our findings are consistent with a scenario in which diverse *spa*-type t008 strains are imported from external reservoirs and subsequently lead to intra-hospital transmission events involving multiple patients, echoing previous observations that this genotype has become a significant cause of healthcare associated infections. Contrastingly, the low nucleotide diversity and higher proportion of imported cases of t045 suggests that a significantly less diverse population exists within a source reservoir that is potentially more localized than the community-wide population of t008. For both t008 and t045, reservoirs may exist within the hospital, healthcare network, community, or as more recently suggests, the household, where parents and caregivers may have contact during the perinatal period [11,24]. Taken together, we find that MRSA importations are a significant contributor to ongoing transmission within the NICU, and as the NICU represents a unique hospital unit in terms of patient population, clinical care, and visitation, tailored interventions to prevent introductions are warranted.

### Population Dynamics of t008 and t045

High-resolution phylogenetic analysis has been applied to understanding the evolution and global spread of MRSA, with applications to investigating hospital transmission [25–27]. This approach involves the assessment of recombination rates, demographic processes (i.e., changes in effective population size), and mutation rates over time. Here, we apply these methods to investigate the population dynamics of MRSA t045 and t008 *spa*-types using the Bayesian coalescent framework. For this analysis, historical t008 (n = 6) and t045 (n = 22) isolates from infants hospitalized in the Hospital-A NICU from 2003–2007 and 2011 were use to extend the longitudinal time-scale and improve molecular clock calibration (S1D Fig). In addition 96 *spa*-type t008 genomes from a previously published sample of multiple healthcare facilities in the same geographical area (S6 Table), were included to assess community level population dynamics (S1E Fig).

We first assessed recombination rates and population structure of t008 and t045 isolates. The average *r/m*, the ratio of SNPs imported through recombination to those occurring through mutation, of the t008 population was 0.98 compared to 0.06 in the t045 population. The recombination rate for the t008 population is slightly higher than the range of values estimated by Enright *et al*. using MLST data [28]; however, remains on the lower end of recombination rates compared to other bacteria [29]. Recombination events were then mapped onto the t045 and t008 phylogenies. In the t045 phylogeny, two clades separated by a deep branch were delineated by unique recombination events, suggesting the presence of two divergent ST225 populations of *spa*-type t045 concurrently circulating (S8 Fig). These two populations were more clearly observed when t045 strains from our study were phylogenetically assessed in the context of other internationally recognized HA- genotypes including the ST225 reference strain (S9 Fig). The t008 phylogeny illustrated a well-supported monophyletic clade comprised largely of Hospital A NICU isolates (77.4% of the total Hospital A NICU sample) that possessed several shared recombination events distinct from the community sample (S10 Fig). The remaining isolates were interspersed among the t008 population concurrently sampled from neighboring hospitals (S10 and S11 Figs). This suggests that the Hospital A NICU MRSA t008 population shares a recent common ancestor distinct from the larger community sample (i.e., the NICU isolates belong to a sub-population drawn for the larger community diversity). Overall, the Bayesian and ML phylogenies illustrates the range of community- and hospital-level diversity of MRSA among individuals seeking care within a defined healthcare region and the variation in diversity between *spa*-type t045 and t008 populations.

**Fig 2:**
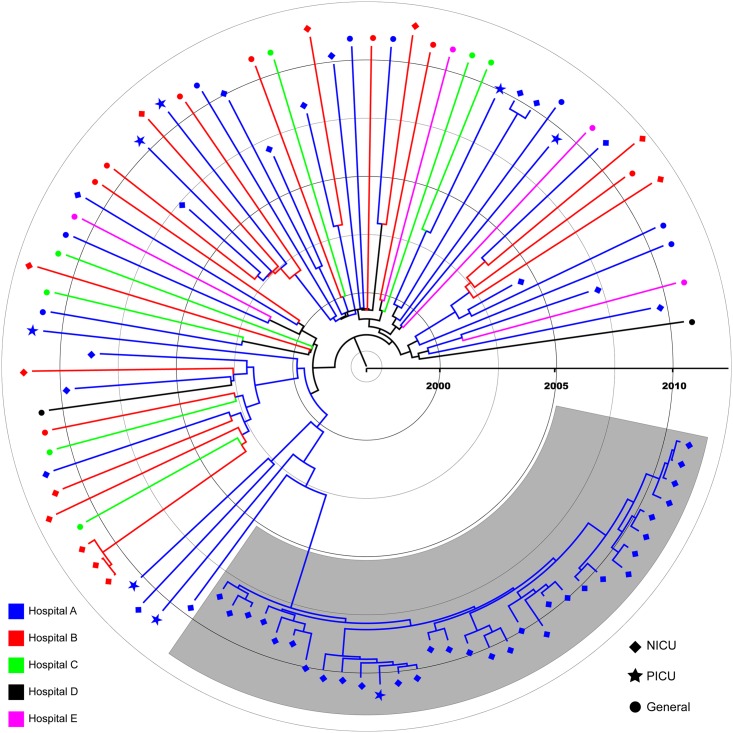
Bayesian maximum clade credibility (MCC) phylogeny of 97 *spa*-type t008 from multiple healthcare facilities including 46 from colonized patients hospitalized in Hospital-A’s NICU (blue branches and diamond tips). The phylogeny is scaled in time with tip dates corresponding to collection dates of positive MRSA cultures. The shaded area represents a monophyletic clade comprised of 31/48 (64.6%) of Hospital-A NICU isolates.

Population dynamics of t045 and t008 *spa-*types were further investigated by assessing tree topology, evolutionary rate, the date of the most recent common ancestor (TMRCA), and changes in *Ne*, a measure of genomic diversity and population growth over time. Bayesian phylogenies rooted by a molecular clock recapitulate the ML analysis of t008 (S12A Fig) and t045 (S12B Fig) populations from Hospital A NICU, highlighting the greater diversity and older ancestry of t008 isolates compared to t045 strains. The evolutionary rate for the community sample of t008 isolates was estimated at ~3.22 (95% HPD, 2.37 to 3.99) SNP year^-1^, significantly faster than the t045 rate of 0.18 (95% HPD, 0.07 to 0.3) (S8 Table and S13 Fig). TMRCA for t045 isolates was dated significantly older than t008 isolates, reflecting the deep branch segregating the two diverging ST225 lineages that likely resulted from two separate local introductions. We subsequently dated each t045 lineage individually by assigning lineage 1 (the top lineage on the MCC tree) to a taxon set in BEAST and enforcing a random local molecular clock. The dating of t045 lineage 1, the most prevalent among t045-colonized patients, was 2004 (95% HPD, 2003 to 2005), supporting a more recent introduction. Interestingly, TMRCA for the community-wide sample of t008 isolates was dated at 1997 (95% HPD, 1994 to 2000), immediately preceding the increased incidence of community-onset MRSA infections observed in national surveillance data (Fig 2).

**Fig 3:**
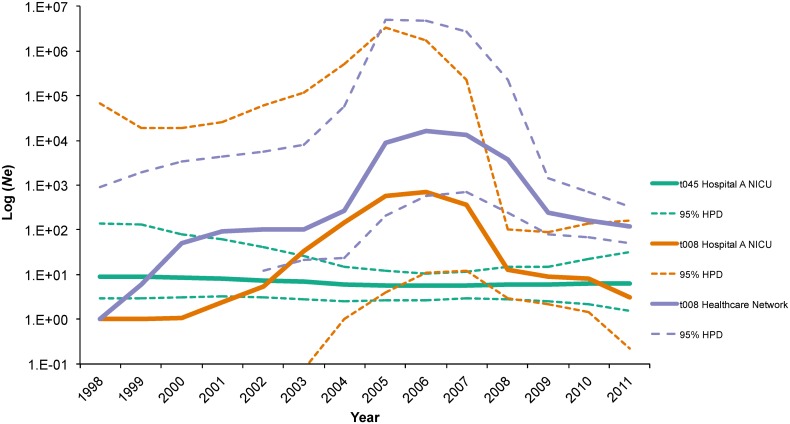
Comparison of effective population sizes (*Ne*) of t045 (green) and t008 (orange) lineages estimated from Bayesian phylogenetic analysis of 46 *spa*-type t008 isolates and 40 *spa*-type t045 isolates from colonized patients hospitalized in Hospital-A’s NICU from 2003–2010 as well as 97 *spa*-type t008 (purple) from multiple healthcare facilities in northeast Florida.

Previous studies have shown variations in bacterial effective population size (*Ne*) during the emergence of successful clones of EMRSA-15 in Singapore [30]; however, similar assessments of the demographic history of USA300/t008 in the healthcare setting are less common. While the t045 *Ne* between 1998 and 2012 remained unchanged (i.e., no population increase or decline), the population of NICU and community samples of t008 isolates experienced rapid population expansion (Fig 3). The beginning of this increase was observed significantly earlier in the healthcare-network sample compared to Hospital-A NICU isolates (Fig 2), supporting the hypothesis that community-level changes in population dynamics drove similar increases among individual hospitals. This observation likely reflects the population level shift between genotypes resulting in the purported displacement of MRSA strains in healthcare settings, including the NICU, which has been observed [8,31–33]. Increased prevalence of MRSA in the community, impacting the proportion of patients, visitors, and healthcare workers with unrecognized MRSA carriage (i.e., colonization pressure) may all have contributed to this displacement [10]. The beginning of the t008 population size increase coincides with both an increase in national outpatient fluoroquinolone prescriptions and the emergence of widespread community-onset MRSA infections in the United States [11]. This observation suggests a larger community epidemic may be associated with population-level evolutionary drivers such as antibiotic use [34]. More recently, the role of household MRSA transmission has been recognized as a driver for the community epidemic, resulting in increased introductions of MRSA into healthcare facilities [11,24]. Certainly, household transmission of t008 could in part explain the multiple introductions observed in our study.

### Comparison of Drug Resistance and Virulence

MRSA genotypes are known to vary genetically in terms of antibiotic resistance and virulence genes as the result of mobile genetic elements and evolutionary history. As the presence or absence of these genes may affect pathogen success, we assessed variations in core and accessory genomes among t008 and t045 isolates (S14 Fig) [35].

Aminoglycoside resistance genes differed between *spa*-types. *Spa*-type t008 strains possessed ant(6)-*la* (*aadE*) and aph(3')-*III* (*aphA-3*), which confer streptomycin and kanamycin resistance [36,37], while t045 strains possessed *aadD* (ant(4')-Ia) and *spc* (transposon Tn554), conferring resistance to a wider range of aminoglycoside antibiotics [36,37]. Macrolide resistance in t045 strains was mediated by *ermA*, while t008 strains possessed macrolide phosphotransferase C (*mphC*) and efflux pump *msrA* [38]. Notably, four t008 strains possessed a 2.4-kb *ermC* carrying plasmid, conferring macrolide and lincosamide resistance [39]. Leukotoxin and Panton-Valentine leucocidin, prototypical of CA-MRSA strains, were common among t008 and t045 isolates. Nine t008 isolates possessed the SaPI5 pathogenicity island carrying *sek2* and *seq2* genes coding for enterotoxins K and Q. Adhesion genes including *ebh*, *fnbA*, *clfA*, and *sdrC/D* were largely present only in t008 isolates (S14 Fig). These virulence genes play a role in host-immune evasion, cell-binding, and, possibly in this case, evolutionary success [40]. Overall, additional research is needed to explore the association of difference.

### Conclusion

Genomic epidemiological studies benefit from the incorporation of pathogen genomic and traditional epidemiological data. In our comprehensive analysis of MRSA colonization risk factors, transmission dynamics, and community-level population dynamics, we show that while patient characteristics are largely similar, transmission varies by strain type. Specifically, for *spa*-type t008, a community-wide epidemic may be driving the importation of strains into the unit, which are then subsequently acquired by susceptible patients. Overall, active surveillance, decolonization, and infection prevention interventions likely contributed to low rates; however, transmission reconstruction elucidated several putative transmission chains resulting from multiple introductions of MRSA into the NICU that would have been impossible to detect by less resolved molecular typing methods. Our analysis should be taken in lieu of specific limitations. First, MRSA surveillance swabs were collected only from the nares of patients, potentially missing colonization at other anatomical sites. Additionally, healthcare workers were not screened during the study, limiting assessment of their potential role in MRSA transmission. For our assessment of transmission within the NICU it was also assumed that decolonization was unsuccessful and patients remained a source of transmission until discharge. Last, for 43.5% of colonized patients, isolates were unavailable for analysis; therefore, unrecognized transmission events may exist.

The current approach to MRSA prevention in the NICU includes identification and elimination of sources for transmission through isolation, cohorting, and/or decolonization of colonized patients [41]. This approach may be ineffective due to lag time between MRSA acquisition and identification of new carrier, when MRSA may be spread [42]. Additionally, only an estimated 41.5% of National Healthcare Surveillance Network hospitals conduct active MRSA surveillance in the NICU [43]. We show that despite a comprehensive prevention program, multiple introductions of t008 and t045 strains contributed significantly to colonization. Therefore, while infection prevention should continue to target horizontal transmission, there is a corresponding need for interventions to mitigate introductions of MRSA into the NICU. This could potentially include routine screening or universal decolonization of parents/caregivers in the prenatal period and a focus on postnatal skin-to-skin contact and parental hygiene [44]. Furthermore, the association between gestational age and t008 colonization may highlight high-risk groups to target these interventions. Clinical trials should further be considered to determine the effectiveness of interventions in preventing the introduction of MRSA into the NICU and reducing infant colonization.

## Methods

### MRSA Surveillance and Study Population

Clinical and demographic information for all hospitalized infants were retrospectively abstracted from electronic medical records. Colonization was defined as a positive surveillance culture, and infection was defined as MRSA isolation from a clinical specimen collected during routine clinical care. Results of weekly MRSA surveillance were made available to the NICU team; however, clinical management of all infants was left to the discretion of the attending neonatologist. Infection prevention and treatment practices followed current guidelines [41,45]. Colonized infants were placed on contact precautions, cohorted, and assigned dedicated clinical staff. Visitors were educated on hand hygiene and contact precautions. Decolonization was attempted using nasal mupirocin, though infants were not rescreened to determine success. Hand hygiene and contact precaution adherence was monitored through infection prevention surveillance and compliance remained high during the study period.

### Sample Selection, *spa*-typing, and Risk Factor Analysis

All available colonization isolates (n = 100) were *spa*-typed as previously described and *spa*-type clustering was assessed through the Based on Repeat Pattern (BURP) algorithm [10,46]. Multivariate linear regression assessed the association between t008 and non-t008 colonization and lengths of stay (LOS). Admission, culture, and discharge dates were used to assess overlapping LOS as well as the daily colonization prevalence. All analyses were performed using R v3.1.1. MRSA isolates identified as *spa*-type t008 (n = 40), representing the most prevalent CA-MRSA genotype, and *spa*-type t045 (n = 16), representing a prevalent HA-MRSA lineage, were selected for WGS (S1C Fig). Additionally, to provide an evolutionary context to the study sample, a convenience sample of “historical” t008 (n = 6) and t045 (n = 22) isolates from infants hospitalized in the NICU from 2003–2007 and 2011 was selected for WGS (S1D Fig). These latter infants were excluded from statistical risk factor comparison of the study population.

### WGS and Phylogenetic Analysis

After ensuring gDNA quality, libraries were prepared using the Illumina Nextera XT library preparation kit and sequenced on the Illumina MiSeq. Sequencing reads were uploaded to NCBI Sequence Read Archive (BioProject PRJNA341781, BioSamples SAMN05728285- SAMN05728425). WGS, SNP calling, and SNP filtering were performed as previously described [10,47]. Paired-end 250 bp reads were filtered using Sickle v1.33, requiring a minimum phred-scaled quality threshold of 20 and minimum read length of 50 bp. *De novo* and reference-based genome assemblies were constructed. *De novo* was performed with SPAdes 3.5 using default kmer sizes and assembled contigs were annotated using Prokka v1.1 [48]. Pangenome analysis of t008 and t045 strains were performed independently using Roary v3.6 and visualized using Phandano (https://jameshadfield.github.io/phandango/) [49]. *De novo* assemblies were also processed through MLST v1.7, ResFinder v2.1, and VirulenceFinder v1.2, to assign MLST profiles and detect antibiotic resistance and virulence genes [50]. For reference-based assemblies, Bowtie2 v2.2.3 was used to map filtered reads of t008 and t045 isolates to MRSA ST8 reference genome USA300 FPR3757 (GenBank accession no. CP000255) and 04–02981 (GenBank accession no. CP001844), respectively. Local realignment around insertions and deletions was performed using GATK v3.6 and recombination events were investigated using Gubbins v2.1.0 [51]. SNPs were called separately for t045 and t008 samples using FreeBayes v0.9.14 requiring a depth of coverage of five and a minimum alternate allele frequency of 0.75. Highly clustered SNPs and SNP locations not conserved across all samples were removed. SNP alignments of *spa*-type t008 and t045 isolates were abstracted from VCF files using a custom python. The final SNP alignment for t008 isolates (n = 46) and t045 isolates (n = 38) contained 486 and 218 SNPs.

To investigate diversity and infer recent transmission event SNP frequencies were compared among t045 and t008 isolates. We applied a recently developed method using a MCMC algorithm to reconstruct transmission trees from epidemiological and genomic data [52]. This approach, implemented in the R package *bitrugs* v0.1, considers both genetic distance between MRSA isolates and temporality of cases, providing an estimation of importations of cases into the NICU as well as acquisitions. Independent runs of 100,000 chain length were performed on t045 and t008 datasets and median parameter values with 95% HPDs were calculated. Transmission networks were reconstructed using the highest probability values for transmission events among patients.

For hospital and healthcare network level diversity, we expanded our *spa*-type t008 Hospital-A NICU sample to include 42 previously published genomes concurrently collected from five hospitals in the local healthcare network (Hospitals A, B, C, D, and E), as well as nine random pediatric intensive care (PICU) surveillance isolates from Hospital-A (S1E Fig and S1 Table) [10]. We then compared diversity between the NICU and community sample by calculating the pairwise genetic distances between all sequenced t008 strains. For a range of genetic distances, we calculated the probability that a pair of strains was sampled from the same location (i.e., NICU or Community).

Furthermore, to understand the phylogenetic relationship of t045 ST-225 strains to other HA-MRSA clones, we compared eight genomes belonging to t002 (ST-5), t003 (ST-225), and t1003 (ST-228) lineages (S9 Fig). All ML phylogenetic analyses were carried out with MEGA v6.06 [53]. The best fitting nucleotide substitution models were selected using the Akaike information criterion. ML phylogenies were then inferred for t008 and t045 isolates using HKY nucleotide substitution model with branching patterns support evaluated by bootstrapping (1000 replicates).

### MRSA Population Dynamics

The demographic histories of *spa*-types t008 and t045 from the Hospital-A NICU and the demographic history of *spa*-type t008 in the local healthcare-network were investigated with BEAST 1.8.0 [47]. The t045 and t008 datasets included colonization isolates from 2003–2011 to provide a more comprehensive estimation of demographic histories. Specifically, the 2003–2011 t008 data set (n = 97), also including the healthcare-network sample, enabled us to track the emergence of *spa*-type t008 among hospitals and the surrounding community. The age for each tip of the phylogeny were represented by the sampling date of positive MRSA surveillance screen from the hospitalized infants. For each MRSA dataset, a posterior distribution of phylogenies was obtained. Evolutionary rates (molecular clock), dating of the most recent common TMRCA, as well as changes in effective population sizes (*Ne*)–a measure of genetic diversity representing the number of genomes effectively contributing to the next generation–were estimated by employing a non-parametric Gaussian Markov randomfield (GMRF) Skygrid evolutionary model [54–57]. For the t045 dataset, relaxed and random-local molecular clock models were compared, assigning all individuals belonging to the lineage 1 monophyletic clade identified in the ML analysis, to a taxa group. This allowed this independent estimation of the TMRCA for each lineage as well as the clock rate to vary across branches. For each dataset, a MCMC was run for 750 million generations with sampling every 75,000. Model mixing was assessed by examination of the effective sampling size (ESS) using Tracer v1.6. Parameter estimates with ESS values of >200 were accepted. Marginal likelihoods estimates for each model were obtained using path sampling and stepping stone analyses and the best fitting models were selected by comparison of Bayes Factors [57–62]. The GMRF Skygrid model enforcing a relaxed molecular clock was selected as the most appropriate representation of the demographic history of t045 and t008 lineages. DensiTree was used to visualize the posterior distribution of trees [63]. The maximum clade credibility (MCC) phylogeny was obtained and statistical support was determined by calculating the posterior probability of each monophyletic clade. DensiTrees and MCC phylogenies were constructed to compare tree topologies between *spa*-types t008 and t045.

### Ethics Statement

The study was approved by the Institutional Review Board (IRB) of the University of Florida (study 2010–059) and reviewed by the Wolfson Children’s Hospital IRB. Isolates and patient data were de-identified and a waiver of consent was obtained.

## Supporting Information

S1 Table Univariate logistic regression of colonization risk factors ordered by statistical significance. (DOCX)

S2 Table Comparison of characteristics between patients with *spa*-typed and non-*spa*-typed isolates ordered by statistical significance. (DOCX)

S3 Table Frequency and proportion of *spa*-types identified among colonized infants in Hospital-A NICU. (DOCX)

S4 Table Multivariate logistic regression of community genotype (*spa*-type *t008*) colonization risk factors ordered by statistical significance. (DOCX)

S5 Table Final multivariate linear regression model of risk factors for increased length of stay among MRSA colonized infants hospitalized in the Hospital-A neonatal intensive care unit (NICU). (DOCX)

S6 Table Frequency of MRSA *spa*-type t008 whole-genome sequences by healthcare facility, hospital unit, and year. (DOCX)

S7 Table Parameter estimates for Markov-chain Monte Carlo (MCMC) analysis of transmission dynamics of *spa*-type t008 and t045 for which whole-genome sequences were available. Beta is the probability that a susceptible patient is colonized given that a colonized patient is present in the unit on that day. Within-cluster diversity is the nucleotide (SNP) diversity within an identified transmission cluster, and between-cluster diversity is the SNP diversity between distinct transmission chains.
(DOCX)

S8 Table Comparison of mean single nucleotide polymorphism (SNP) differences, evolutionary rate, the most recent common ancestor (TMRCA) of *spa*-type t045 isolates from Hospital-A neonatal intensive care unit (NICU) and *spa*-type t008 isolates from Hospital-A NICU and community sample. (DOCX)

S1 Fig Diagram of study population, data sets, and analyses. Data sets are labeled A-E and correspond to specified analyses.
(PDF)

S2 Fig Patient colonizations by MRSA *spa*-type and day. The upper half of the figure represents the date of admission (beginning of grey bar) and date of positive MRSA result (black diamond) until discharge for *spa*-type t008 (orange) and t045 (dark grey) MRSA colonized patients whose isolates were sequenced. The bottom half represents the daily prevalence of colonized patients in Hospital-A NICU, assuming that patients remained colonized from the date of positive surveillance culture until discharge. Among colonized patients, there are few periods when a *spa*-type t008 colonized infant is not present in the Hospital-A NICU. It is possible that this is one ongoing transmission chain. However, among *spa*-type t045 colonized infants, there are at least four discrete periods when colonized infants are present.
(PDF)

S3 Fig Based Upon Repeat Pattern (BURP) clustering of MRSA *spa*-types of 100 isolates from colonized infants in Hospital-A neonatal intensive care unit (NICU). The size of the circle represents the proportion of isolates within each *spa*-type. Non-clustering singleton *spa*-types were excluded from the figure.
(PDF)

S4 Fig Comparison of the pairwise SNP difference frequencies of MRSA genomes between neonatal intensive care unit (NICU) patients colonized with A.) t008 isolates and B.) t045 isolates. (PDF)

S5 Fig Comparison of the pairwise SNP difference frequencies between neonatal intensive care unit (NICU) and community sample of *spa*-type t008 isolates. The comparison is made through assessing SNPs in the core genomes of 97 *spa*-type t008 isolates from five healthcare facilities in northeast Florida, including 46 isolates from Hospital-A NICU (Blue) and 50 isolates from four other facilities (Red). The isolates from other facilities represent the community-level diversity of MRSA and provide a benchmark for the comparison of the epidemiological relatedness (i.e., recent vs distant transmission events) of strains.
(PDF)

S6 Fig Population structure of t008 isolates from the community sample and Hospital A NICU. The pairwise genetic distances calculated as the frequency of SNPs between all sequenced t008 strains were computed. The black dots represent the proportion of pairwise comparisons (y-axis) between strains sampled from the same location (i.e. NICU/NICU or Community/Community) for a range of genetic distances (x-axis). One hundred permutations were performed to assess significance by shuffling the sampling locations among isolates. These permutations are represented by the red points.
(PDF)

S7 Fig Transmission networks of t008 and t045 isolates collected from infants hospitalized from 2008–2010. Transmission networks were inferred from MCMC analysis of whole-genome sequencing and epidemiological data (i.e., dates of admission, colonization, and discharge) of all patients hospitalized in the NICU from 2008–2010. Nodes represent individual infants and directed links represent inferred transmission events. Nodes are labeled with the probability that a case was imported (i.e., not the result of a transmission event in the NICU), and links are labeled with the probability of the inferred transmission event (blue). Links with dashed lines represent putative transmission events that were not statistically significant. While in some instance, multiple transmission routes were possible (i.e., the source on colonization for a patient may have been equally statistically probable from two patients), the links represent the putative transmission event with the highest probability. Therefore the network displays the *best* transmission tree. Transmission clusters are numbered and colored corresponding Fig 1. A) Transmission network of 40 *spa*-type t008 among which 9 [95% HPD: 6–12] importations and 31 [95% HOD: 27–33] acquisitions were inferred. B) Transmission network of 16 *spa*-type t045 isolates among which 8 [95% HPD: 8–10] importations and 8 [95% HPD: 6–8] acquisitions were inferred.
(PDF)

S8 Fig Recombination analysis using Gubbins of *spa*-type t045 (n = 38) isolates collected between the 2003–2011. A) Maximum likelihood phylogeny of 38 isolates inferred using RAxML v8.0.0 using sites nucleotide substitutions not introduced through recombination. B) Simplified version of the 04–02981 reference genome with sites of significant recombination events labeled as follows: 1. Ser-Asp rich fibrinogen-binding (SA2981_0539), 2. non-coding, 3. hypothetical protein (SA2981_2412). C) The center panel depicts recombination events with the rows corresponding to the location in the genome for each tip-label in the maximum likelihood phylogeny. Recombination events colored in red are shared by more than one isolate, while those in blue are unique to an isolate.
(PDF)

S9 Fig Global phylogeny of common healthcare-associated MRSA genotypes and t045 (ST-225) isolates from Hospital-A. NICU groups 1 and 2 correspond to Fig 3B. Genomes in this phylogeny include 04–02981 (NC_017340), ECT-R 2 (FR714927), N315 (NC_002745), 18583 (HE579073), Mu50 (NC_002758), Mu3 (NC_009782), ED98 (NC_013450), CBD-635 (ASHS00000000). *De novo* assemblies of t045 (ST-225) isolates from Hospital-A NICU were aligned to comparison genomes using ProgressiveMauve. Single nucleotide polymorphisms (SNPs) were extracted from homologous regions of the genome. A maximum likelihood phylogeny was inferred using Mega v6.0.6 using GTR nucleotide substitution model with 100 bootstrap replicates. Bootstrap support was 100% for all branches with the exception of the labeled polytomy.
(PDF)

S10 Fig Recombination analysis using Gubbins of *spa*-type t008 (n = 97) isolates collected between the 2003–2011. A) Maximum likelihood phylogeny of 97 isolates inferred using RAxML v8.0.0 using sites nucleotide substitutions not introduced through recombination. B) Simplified version of the USA300 FPR3757 reference genome with sites of significant recombination events labeled as follows: 1. Serine-aspartate repeat containing protein D (WP_000934424), 2. Undecaprenyl-diphosphatase (WP_000469890), 3. mannosyl-glycoprotein endo-beta-N-acetylglucosamidase (WP_000247465), membrane protein (WP_000681154), cell division protein FstK (WP_001251211) and ATPase (WP_001049364), 4. hypothetical protein (WP_001037045), 5. quinone oxidoreductase (WP_001789170), 6. hypothetical protein (WP_001791767), and 7. two-component system response regulator (WP_000697886). C) The center panel depicts recombination events with the rows corresponding to the location in the genome for each tip-label in the maximum likelihood phylogeny. Recombination events colored in red are shared by more than one isolate, while those in blue are unique to an isolate.
(PDF)

S11 Fig Maximum likelihood (ML) phylogeny of 97 *spa*-type t008 isolates from five healthcare facilities in northeast Florida, including 46 isolates from Hospital-A neonatal intensive care unit (NICU), nine isolates from Hospital-A pediatric intensive care unit (PICU), eight from adult hospital associated with Hospital-A NICU, 12 isolates from Hospital-A NICU, and 9, 7, 2, and 4 from Hospitals A-E respectively. Tip labels are colored corresponding to healthcare facilities and branches are scaled in SNPs per site. Asterisks represent clades with bootstrap support values above 80%. The shaded area represents a monophyletic clade comprised of 31/48 (64.6%) of Hospital-A NICU isolates.
(PDF)

S12 Fig DensiTree visualizations of posterior distributions of trees obtained from Bayesian phylogenetic analysis of t008 and t045 datasets using the GMRF skygrid model and relaxed molecular clock as implemented in BEAST v1.8.0. Tip dates are assigned to each node based on the date of collection of positive MRSA surveillance swab, allowing the phylogeny to be scaled in time. The frequency of node clustering is used to assess statistical support for clades, and well-supported branches are indicated by solid colors. A) DensiTree of 46 *spa*-type t008 isolates from colonized patients hospitalized in the NICU of Hospital-A from 2003–2010. B) DensiTree of 40 *spa*-type t045 isolates from colonized patients hospitalized in the NICU of Hospital-A from 2005–2010.
(PDF)

S13 Fig Comparison of evolutionary rates and 95% highest posterior density (HPD) (credibility intervals) of t045 and t008 lineages estimated from Bayesian phylogenetic analysis. Sample includes 46 *spa*-type t008 isolates and 40 *spa*-type t045 isolates from colonized patients hospitalized in Hospital-A NICU from 2003–2010 as well as 97 *spa*-type t008 isolates (Community) from multiple healthcare facilities including 46 from colonized patients hospitalized in Hospital-A NICU.
(PDF)

S14 Fig Bayesian maximum clade credibility (MCC) phylogenies and genotypic antibiotic resistance and virulence identified by ResFinder and VirulenceFinder. Phylogenies were inferred using BEAST v1.8.0 and heatmap was constructed in R v3.2.3. BEAST MCC phylogenies are scaled in time with tip dates corresponding to collection dates of positive MRSA cultures. A) MRSA *spa*-type t008. B) MRSA *spa*-type t045. *Spa*-type t008 strains possessed ant(6)-*la* (previously referred to as *aadE*) and aph(3')-*III* (previously referred to as *aphA*-3), which confer streptomycin and kanamycin resistance, while t045 strains possessed *aadD* (previously referred to as *ant*(4')-Ia) and *spc* (transposon Tn554). Macrolide resistance in t045 strains was mediated by *ermA*, while t008 strains possessed macrolide phosphotransferase C (*mphC*) and efflux pump *msrA*. As a note, gene identification required a 98% sequence indentification; therefore, absence of genes may be due in part to limitations in *de novo* genome assembly.
(PDF)
