# Supplementary material for: Genomic Epidemiology of Methicillin-Resistant Staphylococcus aureus in a Neonatal Intensive Care Unit
Source: PLoS One. 2016 Oct 12;11(10):e0164397. doi: 10.1371/journal.pone.0164397 (PMC5061378; doi:10.1371/journal.pone.0164397)
Supplement: S1 Table — (DOCX) [file pone.0164397.s001.docx]

| Variable (reference) | Odds-Ratio and 95% CI | p-value |
| --- | --- | --- |
| Birth weight by 1 kg | 0.44 (0.36-0.53) | <0.001 |
| Gestational age by 1 week | 0.84 (0.81-0.87) | <0.001 |
| Birth by caesarean section (vaginal) | 1.71 (1.22-2.44) | 0.002 |
| Black race (white) | 1.72 (1.22-2.39) | 0.002 |
| Born off-site (inborn) | 0.59 (0.41-0.85) | 0.005 |
| Other race (white) | 1.58 (0.82-2.82) | 0.14 |
| Multiple births | 1.27 (0.88-1.81) | 0.19 |
| Gender (male) | 1.12 (0.82-1.53) | 0.47 |
