# Supplementary material for: Genomic Epidemiology of Methicillin-Resistant Staphylococcus aureus in a Neonatal Intensive Care Unit
Source: PLoS One. 2016 Oct 12;11(10):e0164397. doi: 10.1371/journal.pone.0164397 (PMC5061378; doi:10.1371/journal.pone.0164397)
Supplement: S2 Table — (DOCX) [file pone.0164397.s002.docx]

|  | *spa*-typed (n=100) | Not *spa*-typed (n=77) | Level of significance (p-value)* |
| --- | --- | --- | --- |
| White race | 53.0% (53/100) | 63.6% (49/77) | 0.11 |
| Length of stay (median and range) | 56 days (6-268 days) | 36 days (1-295 days) | 0.11 |
| Days to positive MRSA (median and range) | 14.5 (2-150) | 12 (0-167) | 0.13 |
| Born off-site | 19.0% (19/100) | 27.3% (21/77) | 0.20 |
| Gestational age (median and range) | 31 weeks (23-42 weeks) | 32 weeks (23-40 weeks) | 0.23 |
| MRSA infection | 16% (16/100) | 22.0% (17/77) | 0.40 |
| Birth weight (median and range) | 1.58 kg (0.54-4 kg) | 1.6 kg (0.46-4.38 kg) | 0.49 |
| Male gender | 52.0% (52/100) | 57.1% (44/77) | 0.59 |
| Birth by caesarean section | 75.0% (75/100) | 71.4% (55/77) | 0.72 |
| Multiple births | 24.0% (24/100) | 24.7% (19/77) | 1.00 |
