# Supplementary material for: Genomic Epidemiology of Methicillin-Resistant Staphylococcus aureus in a Neonatal Intensive Care Unit
Source: PLoS One. 2016 Oct 12;11(10):e0164397. doi: 10.1371/journal.pone.0164397 (PMC5061378; doi:10.1371/journal.pone.0164397)
Supplement: S3 Table — (DOCX) [file pone.0164397.s003.docx]

| *spa*-type | Frequency (percent) |
| --- | --- |
| t008 | 54 (54.0) |
| t045 | 22 (22.0) |
| t002 | 7 (7.0) |
| t5160 | 3 (3.0) |
| t019 | 2 (2.0) |
| t214 | 2 (2.0) |
| t4554 | 2 (2.0) |
| t922 | 2 (2.0) |
| t148 | 1 (1.0) |
| t363 | 1 (1.0) |
| t330 | 1 (1.0) |
| t586 | 1 (1.0) |
| t711 | 1 (1.0) |
| t14545 | 1 (1.0) |
| Total | 100 (100) |
