# Supplementary material for: Genomic Epidemiology of Methicillin-Resistant Staphylococcus aureus in a Neonatal Intensive Care Unit
Source: PLoS One. 2016 Oct 12;11(10):e0164397. doi: 10.1371/journal.pone.0164397 (PMC5061378; doi:10.1371/journal.pone.0164397)
Supplement: S4 Table — (DOCX) [file pone.0164397.s004.docx]

| Variable (reference) | Odds-Ratio and 95% CI | p-value |
| --- | --- | --- |
| Gestational age by 1 week | 0.79 (0.62-0.99) | 0.05 |
| Multiple births | 0.38 (0.12-1.10) | 0.08 |
| Black race (white) | 0.55 (0.20-1.46) | 0.24 |
| Other race (white) | 2.26 (0.51-12.38) | 0.30 |
| Days to positive MRSA | 0.99 (0.97-1.01) | 0.32 |
| Born off-site | 1.75 (0.56 5.82) | 0.35 |
| Birth by caesarean section (vaginal) | 1.56 (0.55-4.49) | 0.41 |
| Birth weight by 1 kg | 1.63 (0.498-5.66) | 0.42 |
| Gender (male) | 1.02 (0.43-2.41) | 0.96 |
