# Supplementary material for: Genomic Epidemiology of Methicillin-Resistant Staphylococcus aureus in a Neonatal Intensive Care Unit
Source: PLoS One. 2016 Oct 12;11(10):e0164397. doi: 10.1371/journal.pone.0164397 (PMC5061378; doi:10.1371/journal.pone.0164397)
Supplement: S5 Table — (DOCX) [file pone.0164397.s005.docx]

| Variable (reference) | Coefficient (95% CI) | p-value |
| --- | --- | --- |
| Days to positive MRSA | 0.84 (0.56-1.12) | <0.001 |
| MRSA Infection (No infection) | 30.00 (13.03-46.97) | <0.001 |
| Gestational age by 1 week | -3.85 (-7.25--0.45) | 0.03 |
| *spa*-type (non-t008) | 8.89 (-3.63-21.17) | 0.16 |
| Birth weight by 1 kg | 1.74 (-15.42-18.89) | 0.84 |
