# Supplementary material for: Genomic Epidemiology of Methicillin-Resistant Staphylococcus aureus in a Neonatal Intensive Care Unit
Source: PLoS One. 2016 Oct 12;11(10):e0164397. doi: 10.1371/journal.pone.0164397 (PMC5061378; doi:10.1371/journal.pone.0164397)
Supplement: S6 Table — (DOCX) [file pone.0164397.s006.docx]

| Healthcare Facility | Unit | 2003 | 2006 | 2007 | 2008 | 2009 | 2010 | 2011 | Total |
| --- | --- | --- | --- | --- | --- | --- | --- | --- | --- |
| Hospital-A | NICU | 1 | 3 | 1 | 8 | 14 | 18 | 1 | 46 |
| Hospital-A | PICU |  |  |  |  | 2 | 7 |  | 9 |
| Hospital-A | General |  |  |  |  |  | 8 |  | 8 |
| Hospital-B | NICU |  |  |  |  |  | 3 | 9 | 12 |
| Hospital-B | General |  |  |  |  |  | 9 |  | 9 |
| Hospital-C | General |  |  |  |  |  | 7 |  | 7 |
| Hospital-D | General |  |  |  |  |  | 2 |  | 2 |
| Hospital-E | General |  |  |  |  |  | 4 |  | 4 |
| Total |  | 1 | 3 | 1 | 8 | 16 | 58 | 10 | 97 |
