# Supplementary material for: Genomic Epidemiology of Methicillin-Resistant Staphylococcus aureus in a Neonatal Intensive Care Unit
Source: PLoS One. 2016 Oct 12;11(10):e0164397. doi: 10.1371/journal.pone.0164397 (PMC5061378; doi:10.1371/journal.pone.0164397)
Supplement: S7 Table — Beta is the probability that a susceptible patient is colonized given that a colonized patient is present in the unit on that day. Within-cluster diversity is the nucleotide (SNP) diversity within an identified transmission cluster, and between-cluster diversity is the SNP diversity between distinct transmission chains. (DOCX) [file pone.0164397.s007.docx]

|  | t008 Isolates (n=40) median (95% HPD) | t045 Isolates (n=16) median (95% HPD) |
| --- | --- | --- |
| Beta (Transmission parameter) | 3.94E-4 [2.64E-4 - 5.49E-4] | 2.99E-4 [1.07E-4 - 5.43E-4] |
| Within-cluster diversity (SNPs) | 3.33 [-0.87 - 47.10] | 0.98 [-0.79 - 6.74] |
| Between-cluster diversity (SNPs) | 26.6 [24.2 - 29.1] | 57.75 [47.5 - 69.9] |
| Importations | 9 [6 - 12] | 8 [8 - 10] |
| Acquisitions | 31 [27 - 33] | 8 [6 - 8] |
