# Supplementary material for: Genomic Epidemiology of Methicillin-Resistant Staphylococcus aureus in a Neonatal Intensive Care Unit
Source: PLoS One. 2016 Oct 12;11(10):e0164397. doi: 10.1371/journal.pone.0164397 (PMC5061378; doi:10.1371/journal.pone.0164397)
Supplement: S8 Table — (DOCX) [file pone.0164397.s008.docx]

| Sample | *spa*-type | SNPs | Mean nucleotide differences | Evolutionary Rate | TMRCA |
| --- | --- | --- | --- | --- | --- |
| Hospital-A NICU | t008 | 482 | 39.5 | 1.58E-06 [95% HPD: 2.30E-06, 8.56E-07] | 2000 [95% HPD: 1995, 2004] |
| Community | t008 | 1928 | 64 | 1.12E-06 [95% HPD: 1.39E-06, 8.26E-07] | 1997 [95% HPD: 1994, 2000] |
| Hospital-A NICU | t045 | 240 | 56.14 | 6.36E-08 [95% HPH: 1.19E-07, 2.54E-08] | 1989 [95% HPD: 1982, 1995] |
