# Supplementary figures and images for: Genomic Epidemiology of Methicillin-Resistant Staphylococcus aureus in a Neonatal Intensive Care Unit
Source: PLoS One. 2016 Oct 12;11(10):e0164397. doi: 10.1371/journal.pone.0164397 (PMC5061378; doi:10.1371/journal.pone.0164397)

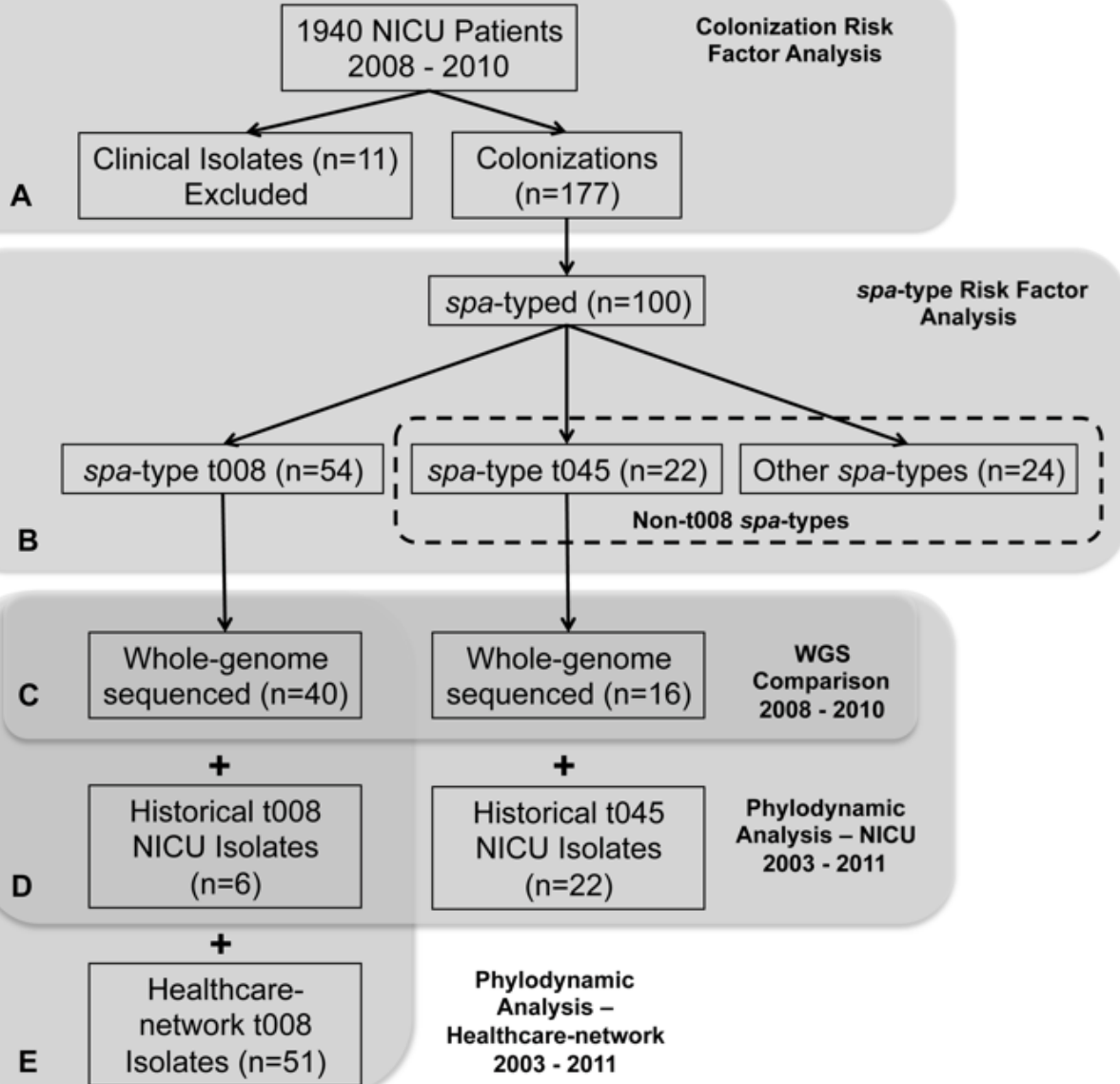

Supplement: S1 Fig — Data sets are labeled A-E and correspond to specified analyses. (PDF) [file pone.0164397.s009.pdf]

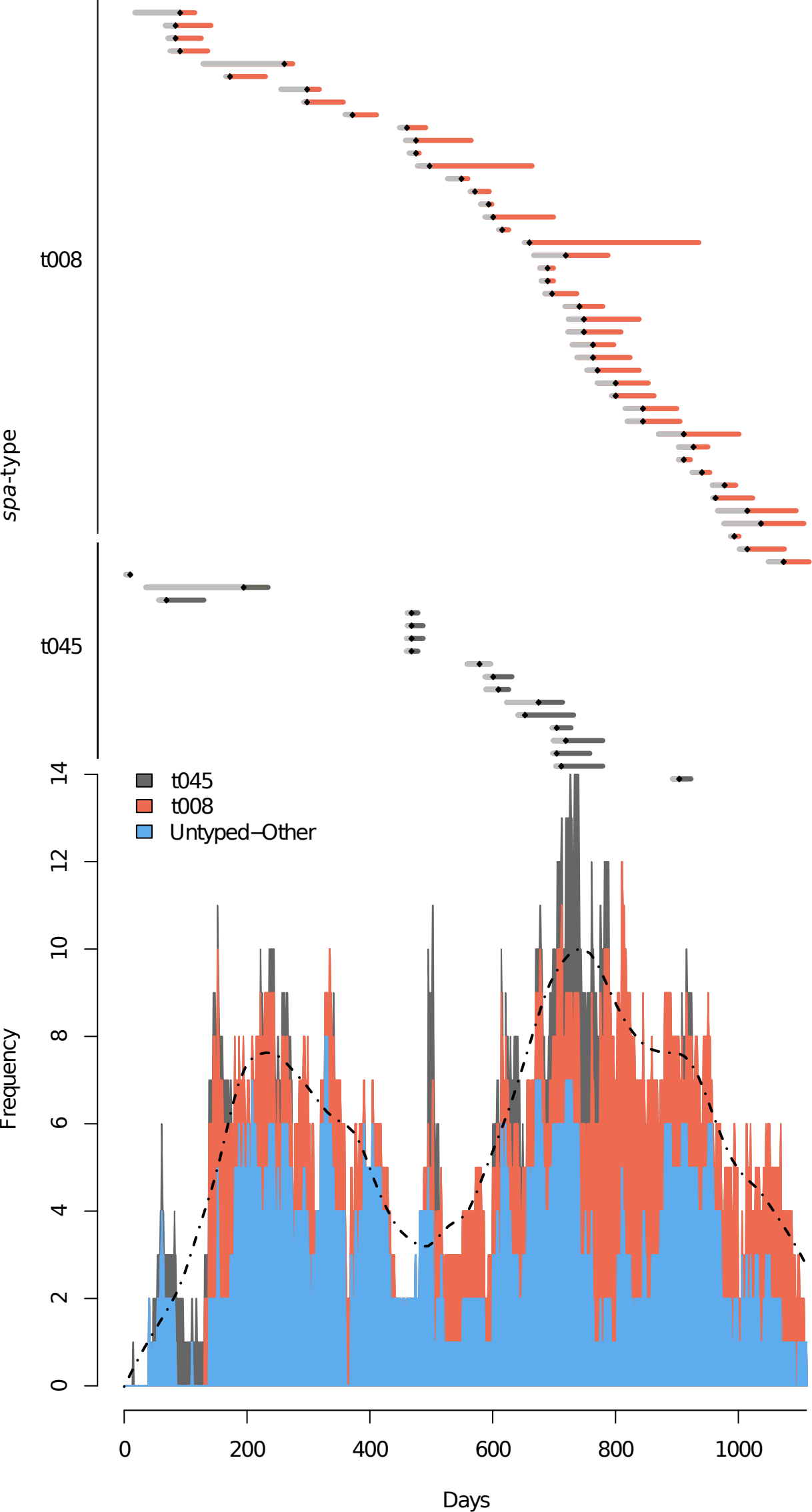

Supplement: S2 Fig — The upper half of the figure represents the date of admission (beginning of grey bar) and date of positive MRSA result (black diamond) until discharge for spa-type t008 (orange) and t045 (dark grey) MRSA colonized patients whose isolates were sequenced. The bottom half represents the daily prevalence of colonized patients in Hospital-A NICU, assuming that patients remained colonized from the date of positive surveillance culture until discharge. Among colonized patients, there are few periods when a spa-type t008 colonized infant is not present in the Hospital-A NICU. It is possible that this is one ongoing transmission chain. However, among spa-type t045 colonized infants, there are at least four discrete periods when colonized infants are present. (PDF) [file pone.0164397.s010.pdf]

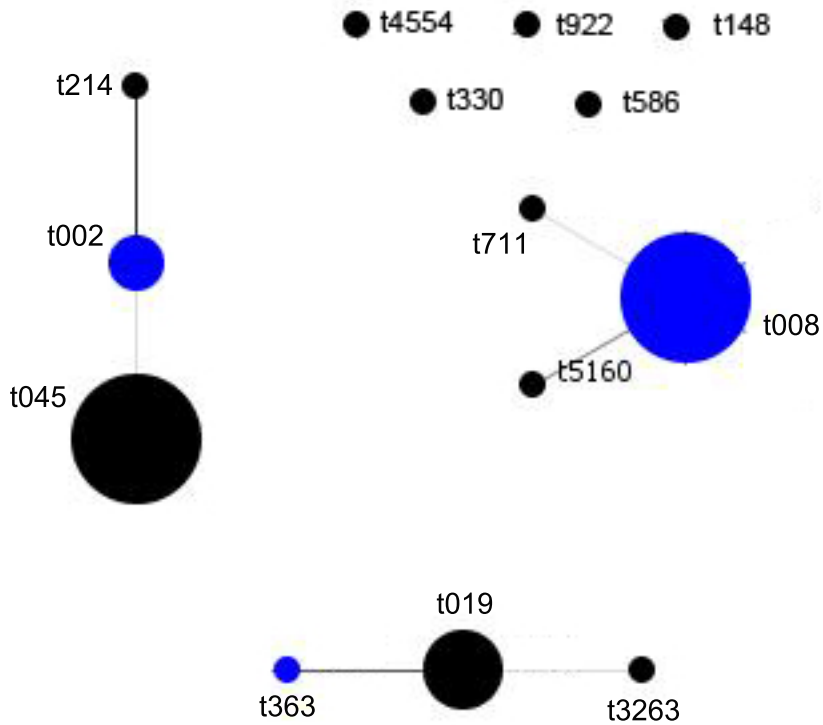

Supplement: S3 Fig — The size of the circle represents the proportion of isolates within each spa-type. Non-clustering singleton spa-types were excluded from the figure. (PDF) [file pone.0164397.s011.pdf]

Distribution of pairwise genetic distances

A.

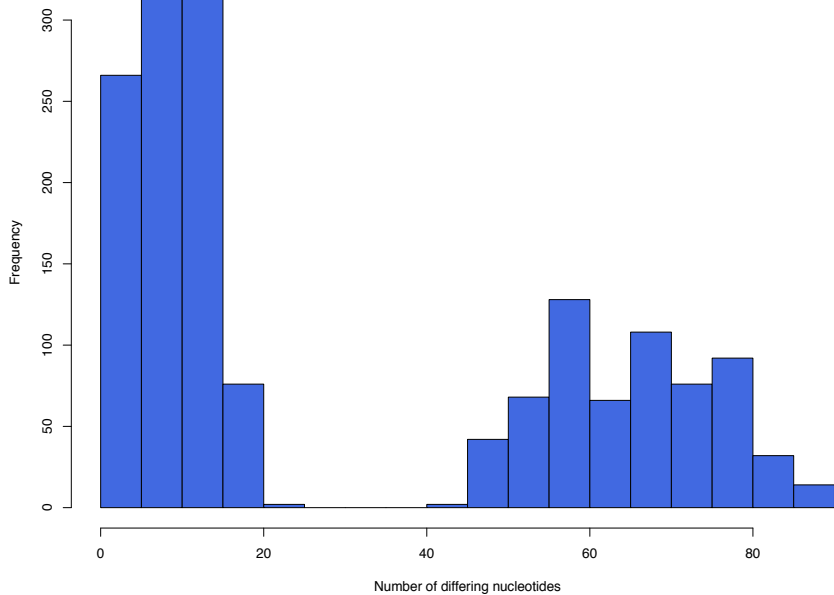

B.

Distribution of pairwise genetic distances

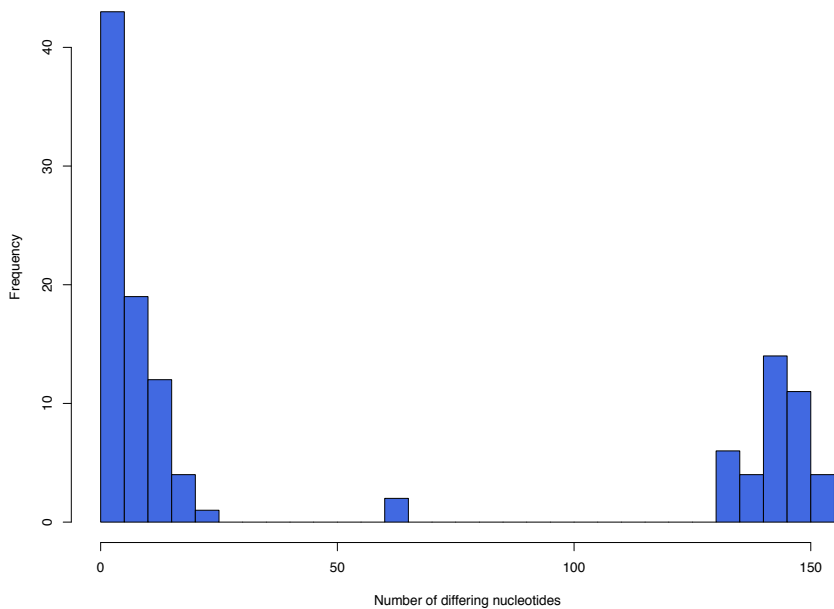

Supplement: S4 Fig — (PDF) [file pone.0164397.s012.pdf]

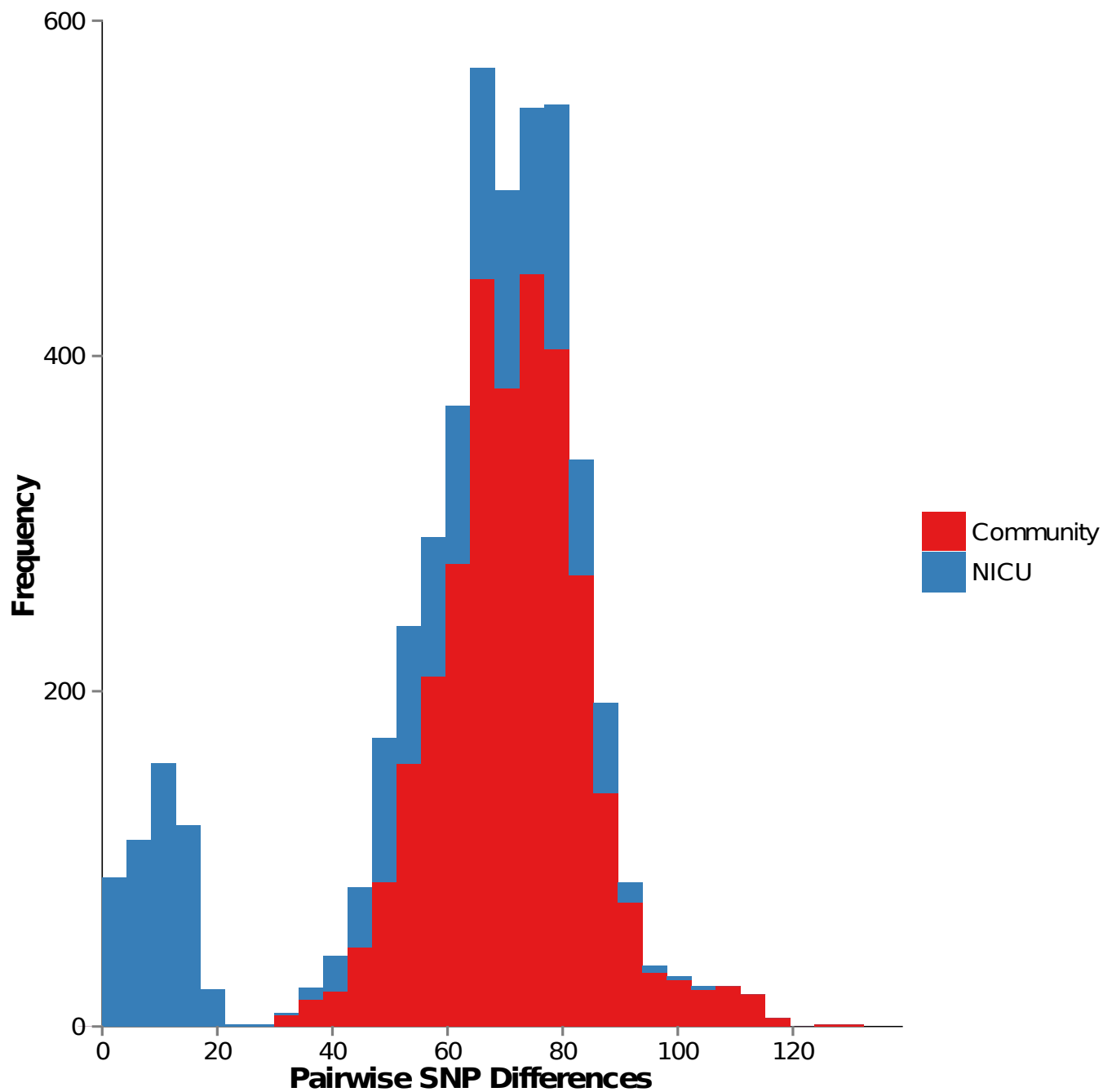

Supplement: S5 Fig — The comparison is made through assessing SNPs in the core genomes of 97 spa-type t008 isolates from five healthcare facilities in northeast Florida, including 46 isolates from Hospital-A NICU (Blue) and 50 isolates from four other facilities (Red). The isolates from other facilities represent the community-level diversity of MRSA and provide a benchmark for the comparison of the epidemiological relatedness (i.e., recent vs distant transmission events) of strains. (PDF) [file pone.0164397.s013.pdf]

# NICU v. Community

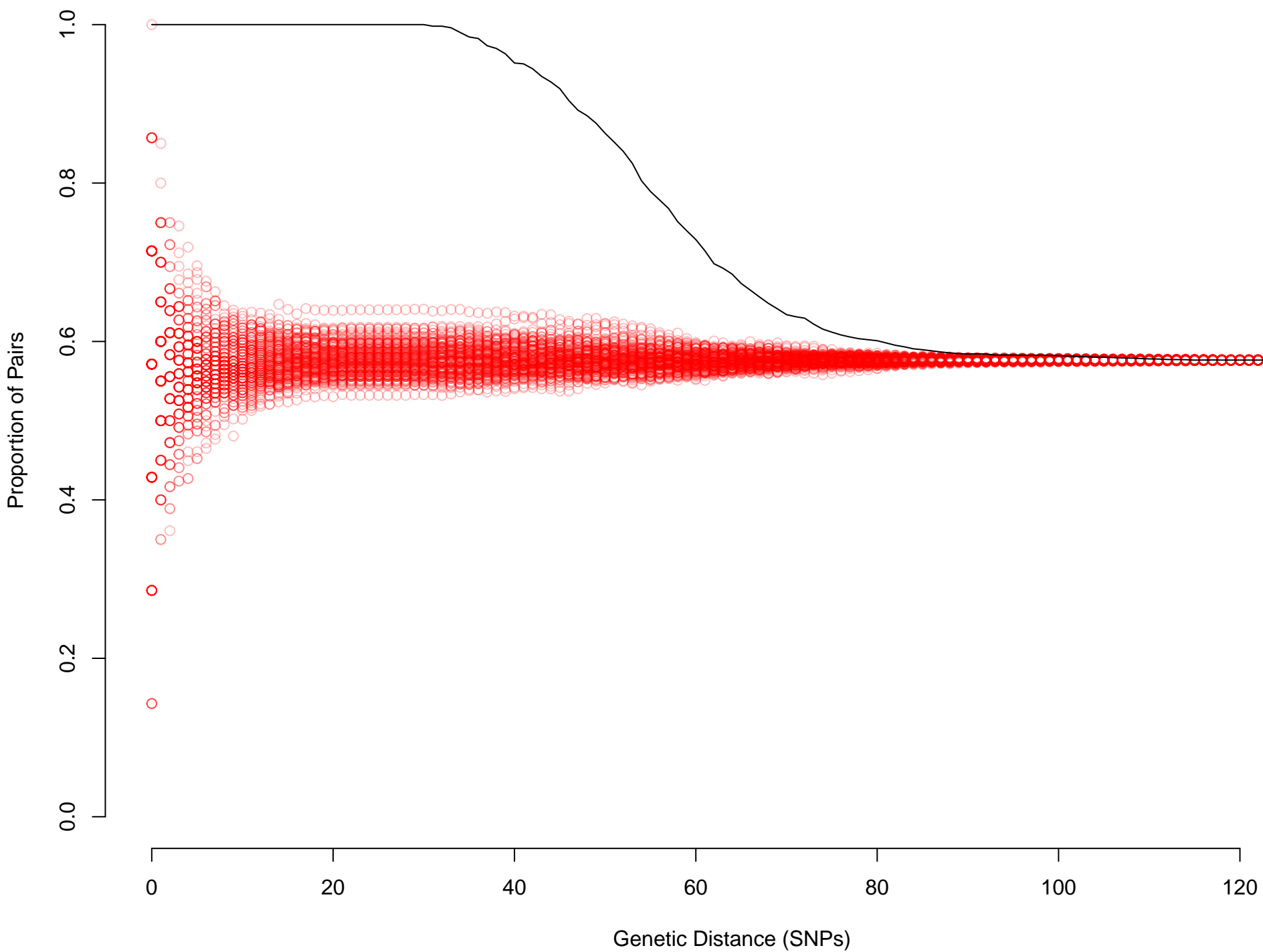

Supplement: S6 Fig — The pairwise genetic distances calculated as the frequency of SNPs between all sequenced t008 strains were computed. The black dots represent the proportion of pairwise comparisons (y-axis) between strains sampled from the same location (i.e. NICU/NICU or Community/Community) for a range of genetic distances (x-axis). One hundred permutations were performed to assess significance by shuffling the sampling locations among isolates. These permutations are represented by the red points. (PDF) [file pone.0164397.s014.pdf]

A.

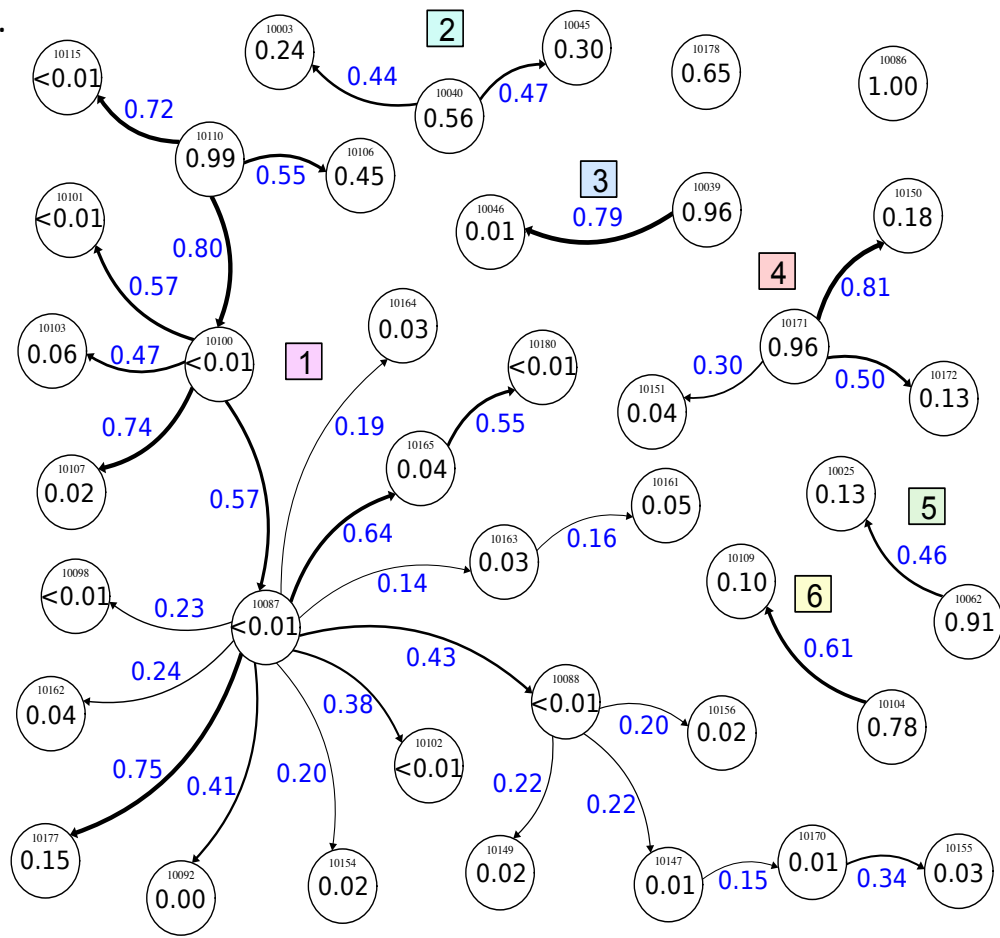

B.

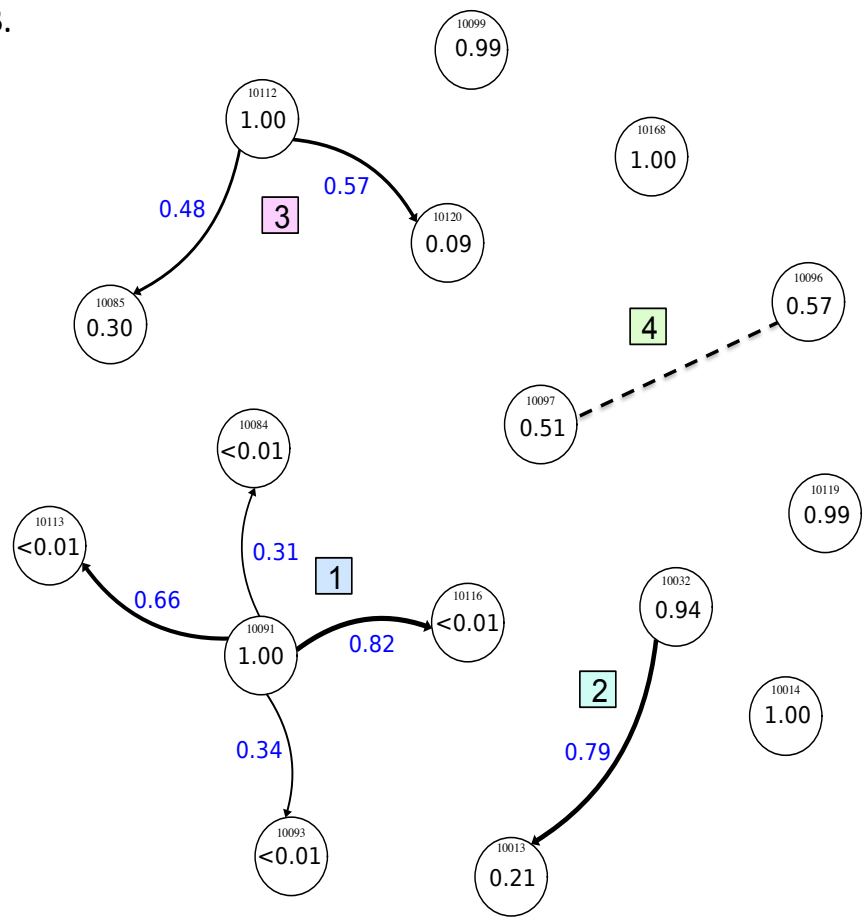

Supplement: S7 Fig — Transmission networks were inferred from MCMC analysis of whole-genome sequencing and epidemiological data (i.e., dates of admission, colonization, and discharge) of all patients hospitalized in the NICU from 2008–2010. Nodes represent individual infants and directed links represent inferred transmission events. Nodes are labeled with the probability that a case was imported (i.e., not the result of a transmission event in the NICU), and links are labeled with the probability of the inferred transmission event (blue). Links with dashed lines represent putative transmission events that were not statistically significant. While in some instance, multiple transmission routes were possible (i.e., the source on colonization for a patient may have been equally statistically probable from two patients), the links represent the putative transmission event with the highest probability. Therefore the network displays the best transmission tree. Transmission clusters are numbered and colored corresponding Fig 1. A) Transmission network of 40 spa-type t008 among which 9 [95% HPD: 6–12] importations and 31 [95% HOD: 27–33] acquisitions were inferred. B) Transmission network of 16 spa-type t045 isolates among which 8 [95% HPD: 8–10] importations and 8 [95% HPD: 6–8] acquisitions were inferred. (PDF) [file pone.0164397.s015.pdf]

A

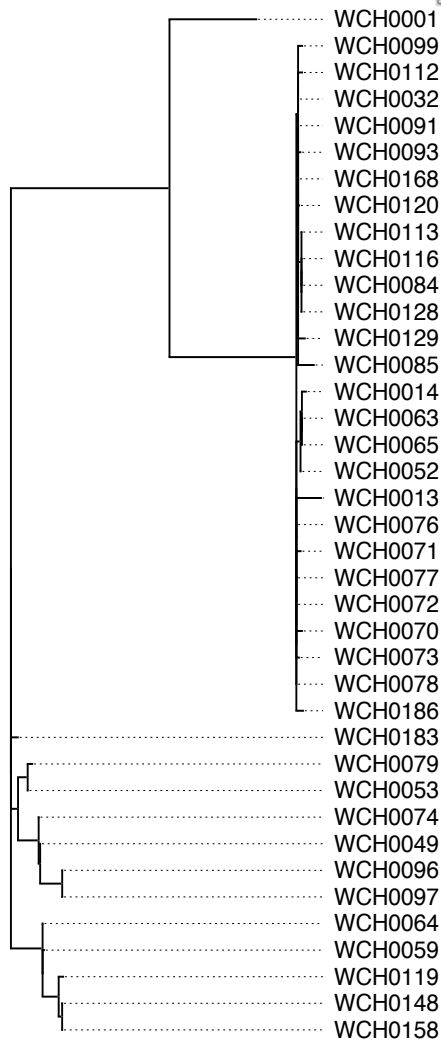

B

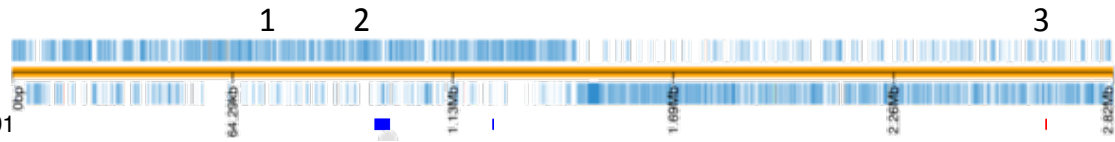

C

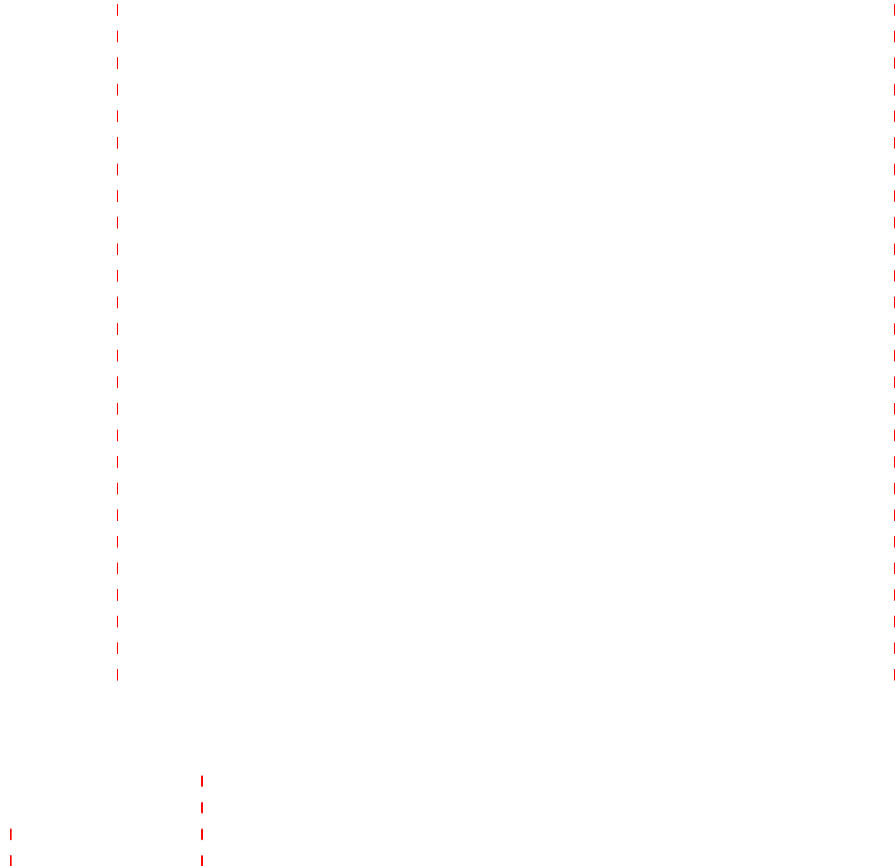

Supplement: S8 Fig — A) Maximum likelihood phylogeny of 38 isolates inferred using RAxML v8.0.0 using sites nucleotide substitutions not introduced through recombination. B) Simplified version of the 04–02981 reference genome with sites of significant recombination events labeled as follows: 1. Ser-Asp rich fibrinogen-binding (SA2981_0539), 2. non-coding, 3. hypothetical protein (SA2981_2412). C) The center panel depicts recombination events with the rows corresponding to the location in the genome for each tip-label in the maximum likelihood phylogeny. Recombination events colored in red are shared by more than one isolate, while those in blue are unique to an isolate. (PDF) [file pone.0164397.s016.pdf]

A

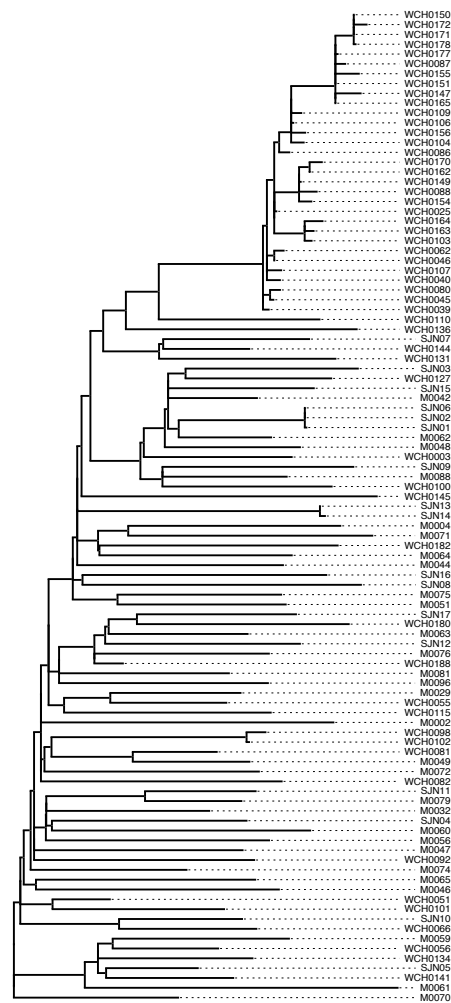

B

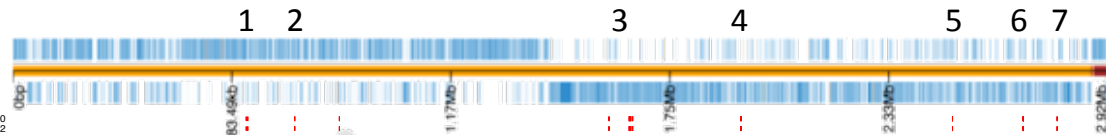

C

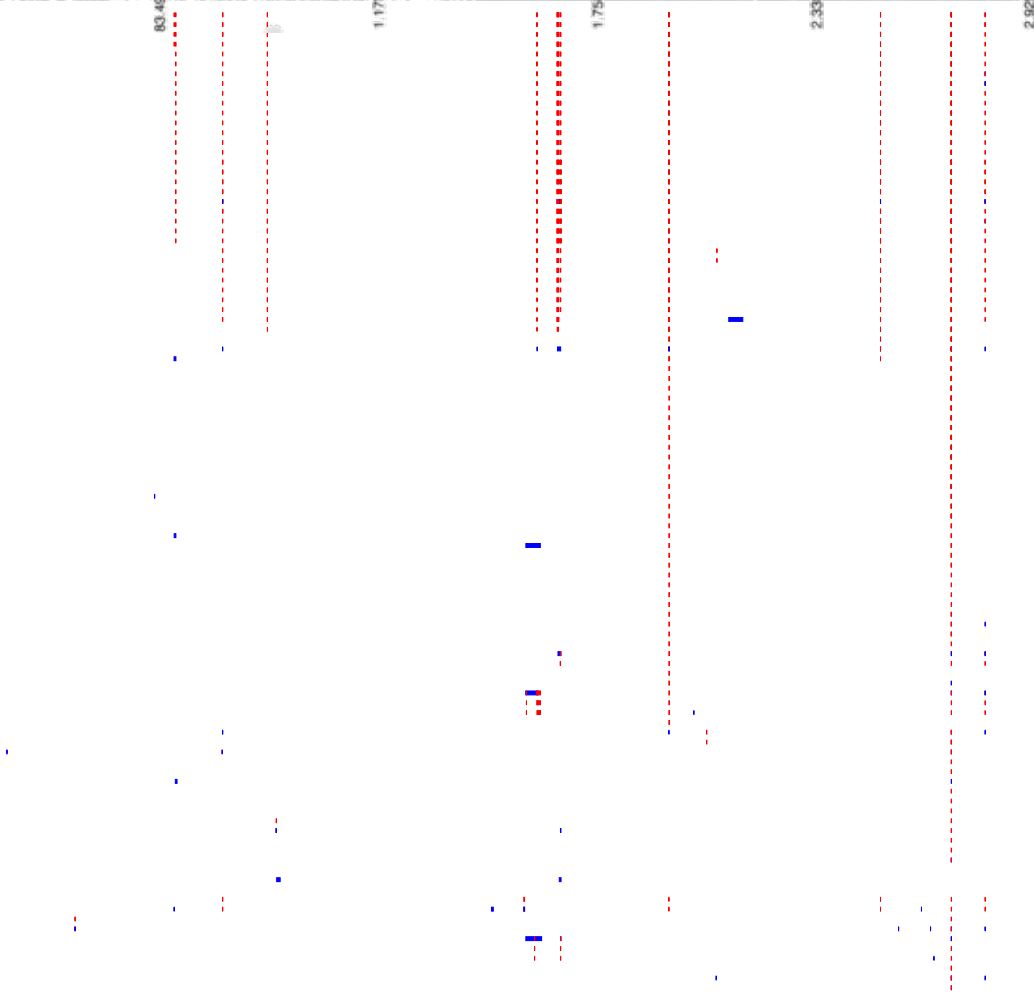

Supplement: S10 Fig — A) Maximum likelihood phylogeny of 97 isolates inferred using RAxML v8.0.0 using sites nucleotide substitutions not introduced through recombination. B) Simplified version of the USA300 FPR3757 reference genome with sites of significant recombination events labeled as follows: 1. Serine-aspartate repeat containing protein D (WP_000934424), 2. Undecaprenyl-diphosphatase (WP_000469890), 3. mannosyl-glycoprotein endo-beta-N-acetylglucosamidase (WP_000247465), membrane protein (WP_000681154), cell division protein FstK (WP_001251211) and ATPase (WP_001049364), 4. hypothetical protein (WP_001037045), 5. quinone oxidoreductase (WP_001789170), 6. hypothetical protein (WP_001791767), and 7. two-component system response regulator (WP_000697886). C) The center panel depicts recombination events with the rows corresponding to the location in the genome for each tip-label in the maximum likelihood phylogeny. Recombination events colored in red are shared by more than one isolate, while those in blue are unique to an isolate. (PDF) [file pone.0164397.s018.pdf]

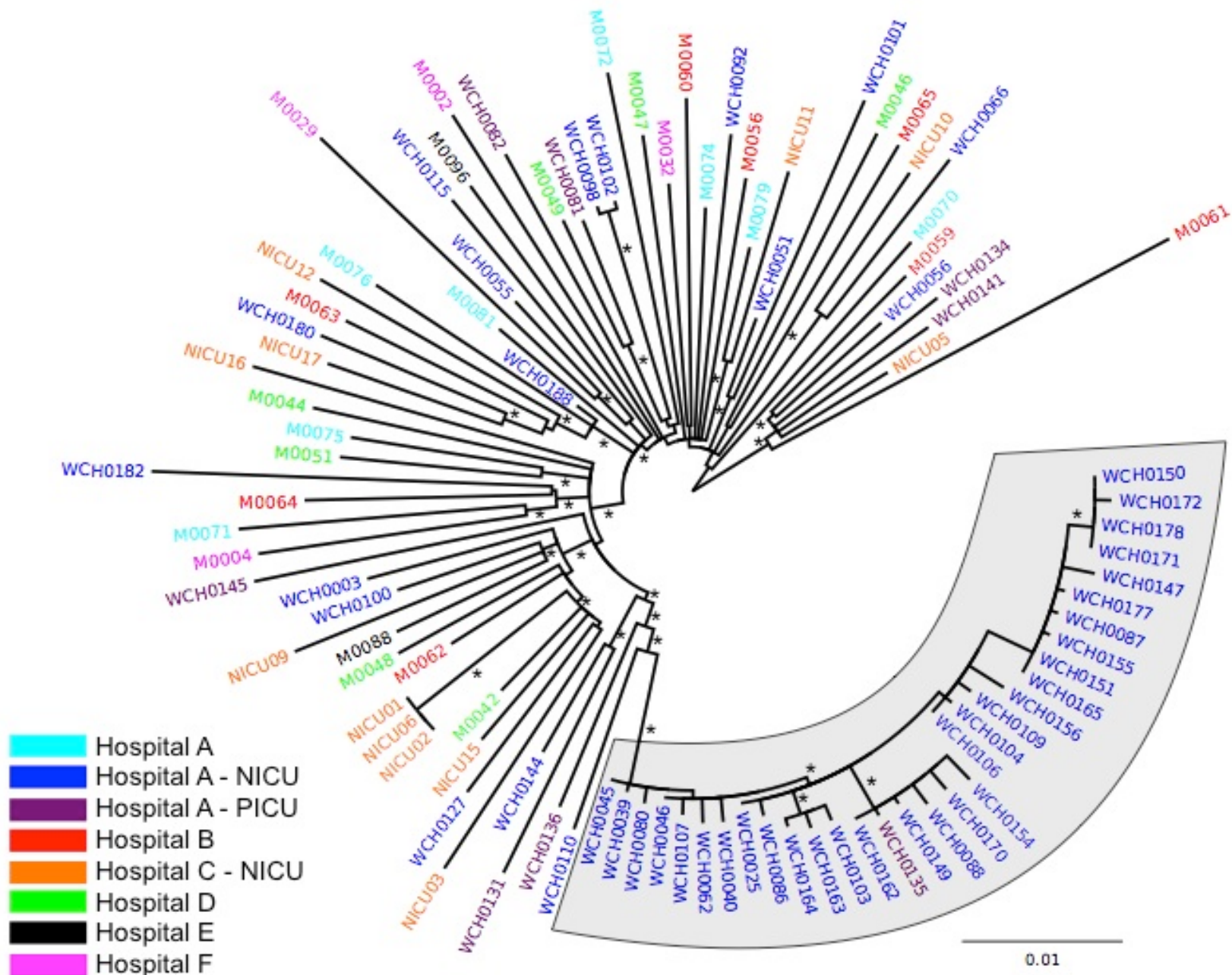

Supplement: S11 Fig — Tip labels are colored corresponding to healthcare facilities and branches are scaled in SNPs per site. Asterisks represent clades with bootstrap support values above 80%. The shaded area represents a monophyletic clade comprised of 31/48 (64.6%) of Hospital-A NICU isolates. (PDF) [file pone.0164397.s019.pdf]

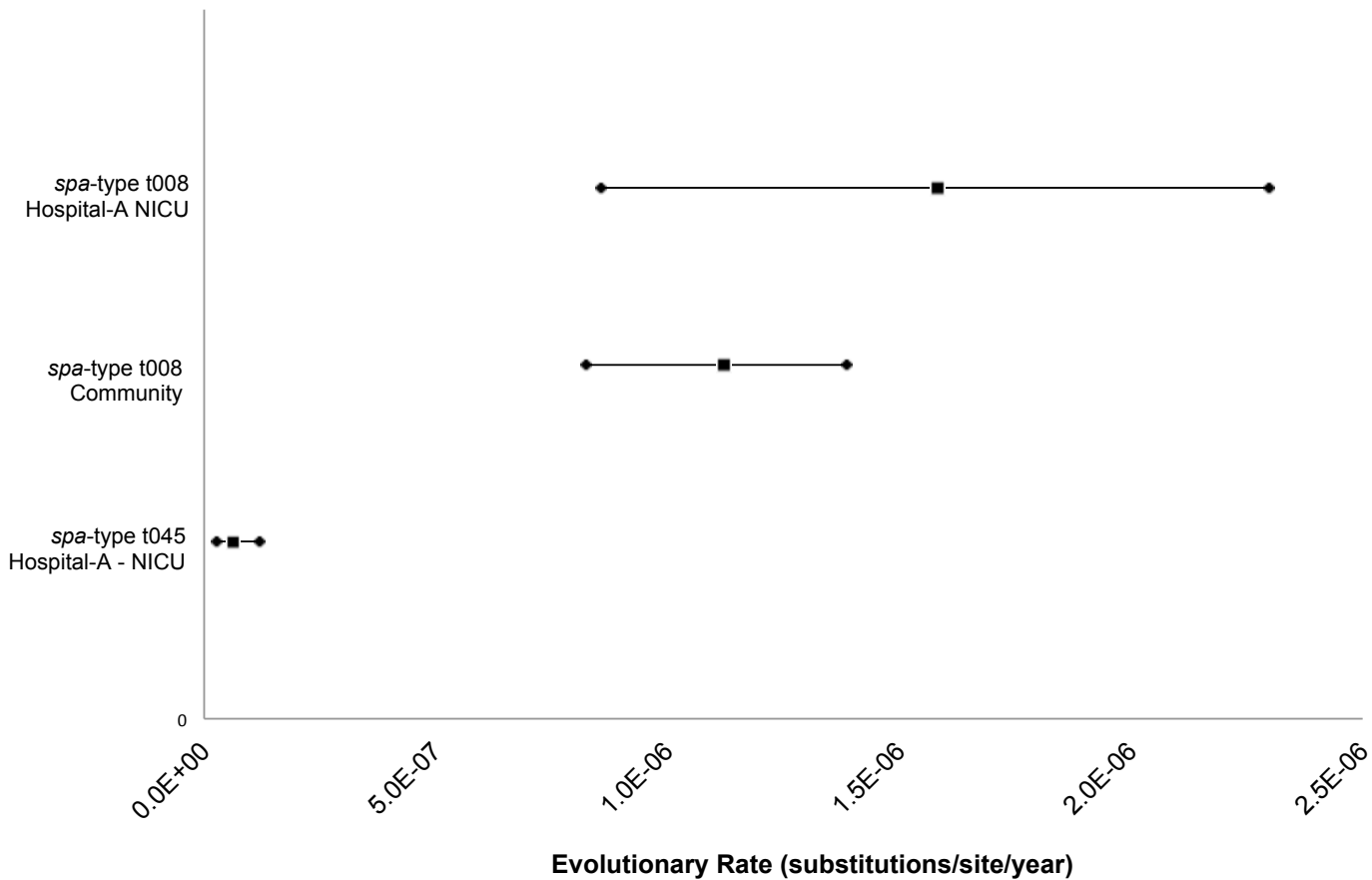

Supplement: S13 Fig — Sample includes 46 spa-type t008 isolates and 40 spa-type t045 isolates from colonized patients hospitalized in Hospital-A NICU from 2003–2010 as well as 97 spa-type t008 isolates (Community) from multiple healthcare facilities including 46 from colonized patients hospitalized in Hospital-A NICU. (PDF) [file pone.0164397.s021.pdf]

A

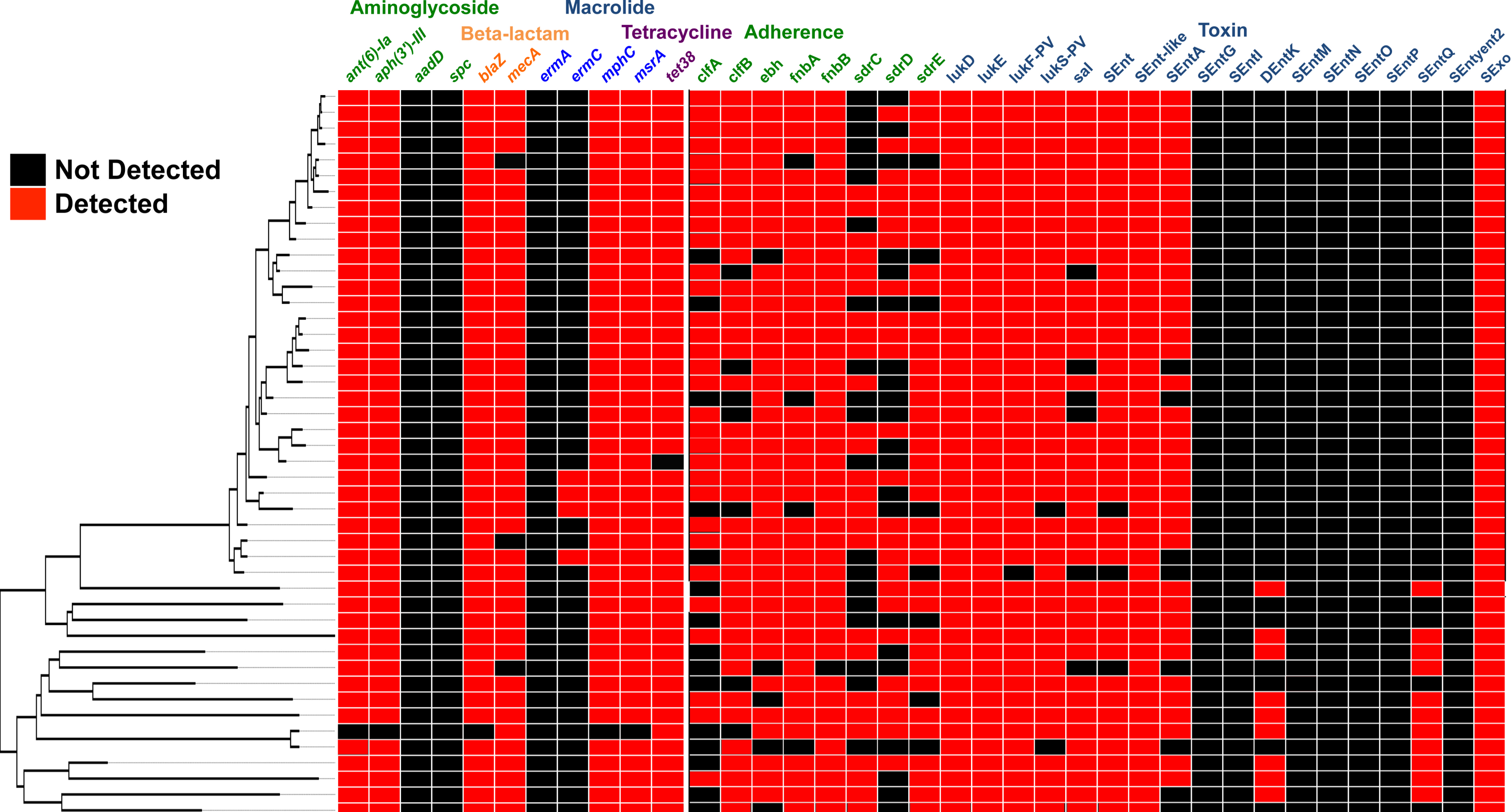

# B

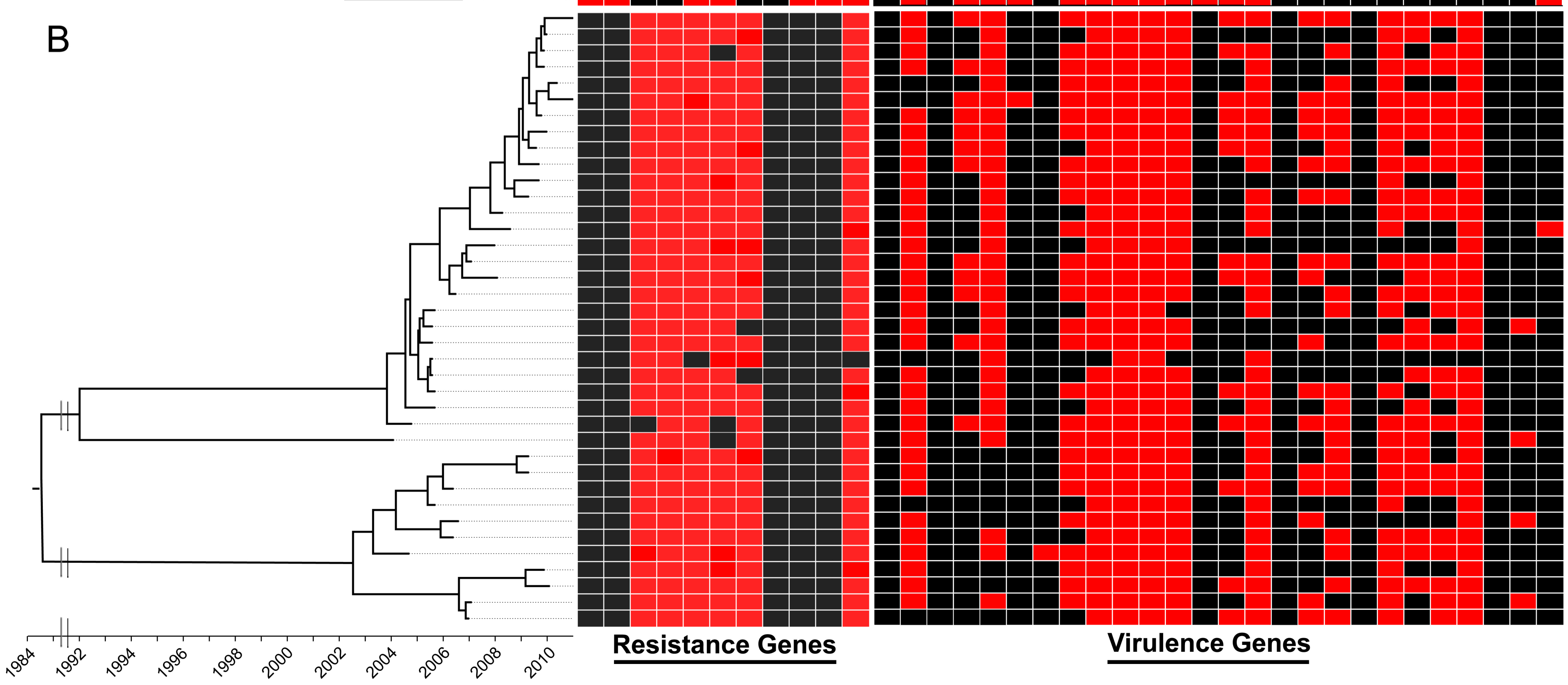

Supplement: S14 Fig — Phylogenies were inferred using BEAST v1.8.0 and heatmap was constructed in R v3.2.3. BEAST MCC phylogenies are scaled in time with tip dates corresponding to collection dates of positive MRSA cultures. A) MRSA spa-type t008. B) MRSA spa-type t045. Spa-type t008 strains possessed ant(6)-la (previously referred to as aadE) and aph(3')-III (previously referred to as aphA-3), which confer streptomycin and kanamycin resistance, while t045 strains possessed aadD (previously referred to as ant(4')-Ia) and spc (transposon Tn554). Macrolide resistance in t045 strains was mediated by ermA, while t008 strains possessed macrolide phosphotransferase C (mphC) and efflux pump msrA. As a note, gene identification required a 98% sequence indentification; therefore, absence of genes may be due in part to limitations in de novo genome assembly. (PDF) [file pone.0164397.s022.pdf]
