# Supplementary material for: Genomic Epidemiology of Methicillin-Resistant Staphylococcus aureus in a Neonatal Intensive Care Unit
Source: PLoS One. 2016 Oct 12;11(10):e0164397. doi: 10.1371/journal.pone.0164397 (PMC5061378; doi:10.1371/journal.pone.0164397)
Supplement: S9 Fig — NICU groups 1 and 2 correspond to Fig 3B. Genomes in this phylogeny include 04–02981 (NC_017340), ECT-R 2 (FR714927), N315 (NC_002745), 18583 (HE579073), Mu50 (NC_002758), Mu3 (NC_009782), ED98 (NC_013450), CBD-635 (ASHS00000000). De novo assemblies of t045 (ST-225) isolates from Hospital-A NICU were aligned to comparison genomes using ProgressiveMauve. Single nucleotide polymorphisms (SNPs) were extracted from homologous regions of the genome. A maximum likelihood phylogeny was inferred using Mega v6.0.6 using GTR nucleotide substitution model with 100 bootstrap replicates. Bootstrap support was 100% for all branches with the exception of the labeled polytomy. (PDF) [file pone.0164397.s017.pdf]

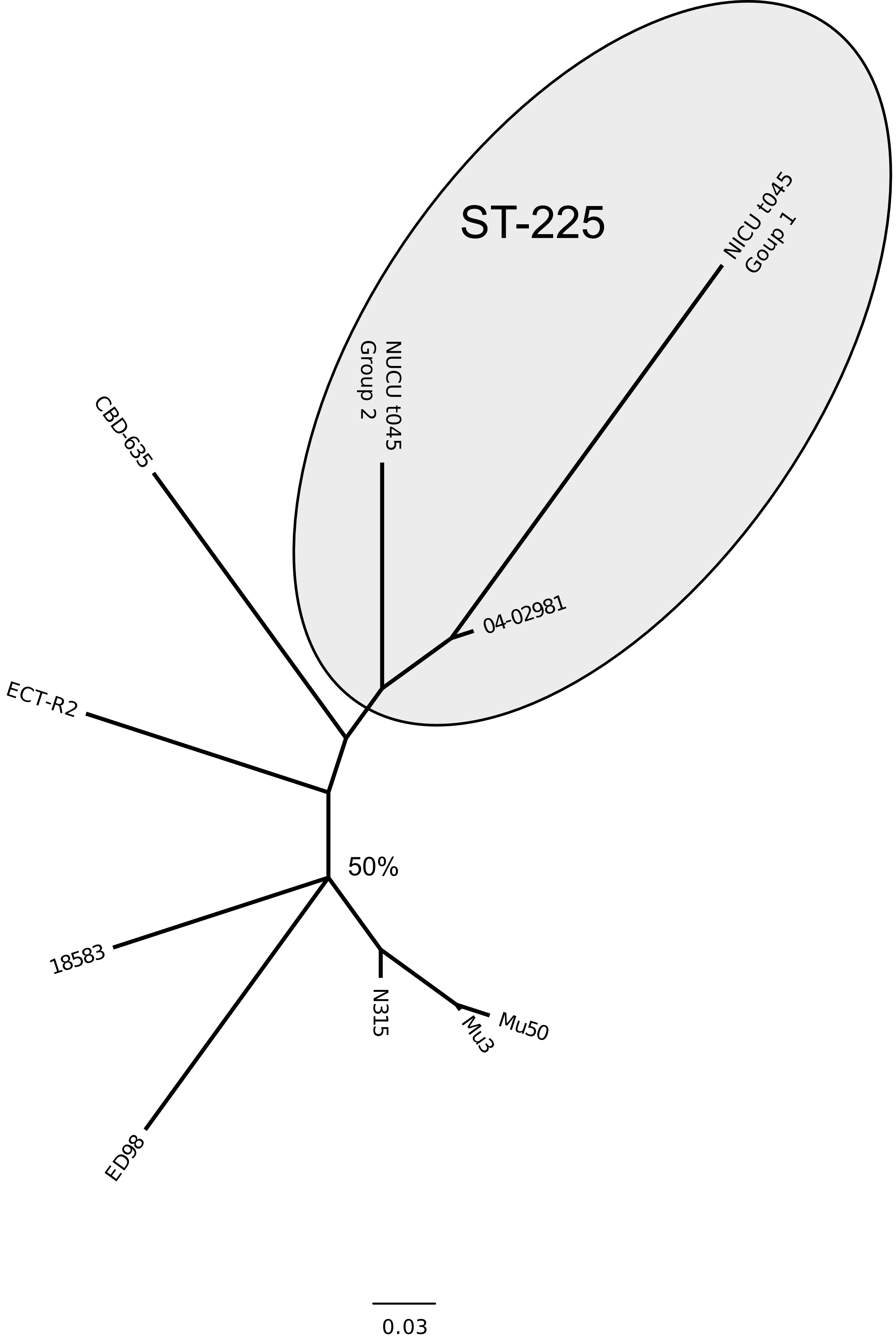

Strain	ispa-type	MLST	Country
04-2981	t003	ST225	Germany
CBD-635	t003	ST5	US
ECT-R 2	t002	ST5	Sweden
18583	t1003	ST228	Germany
ED98	t002	ST5	Ireland
N315	t002	ST5	Japan
Mu50	t002	ST5	Japan
Mu3	t002	ST5	Japan
