# Supplementary material for: Genomic Epidemiology of Methicillin-Resistant Staphylococcus aureus in a Neonatal Intensive Care Unit
Source: PLoS One. 2016 Oct 12;11(10):e0164397. doi: 10.1371/journal.pone.0164397 (PMC5061378; doi:10.1371/journal.pone.0164397)
Supplement: S12 Fig — Tip dates are assigned to each node based on the date of collection of positive MRSA surveillance swab, allowing the phylogeny to be scaled in time. The frequency of node clustering is used to assess statistical support for clades, and well-supported branches are indicated by solid colors. A) DensiTree of 46 spa-type t008 isolates from colonized patients hospitalized in the NICU of Hospital-A from 2003–2010. B) DensiTree of 40 spa-type t045 isolates from colonized patients hospitalized in the NICU of Hospital-A from 2005–2010. (PDF) [file pone.0164397.s020.pdf]

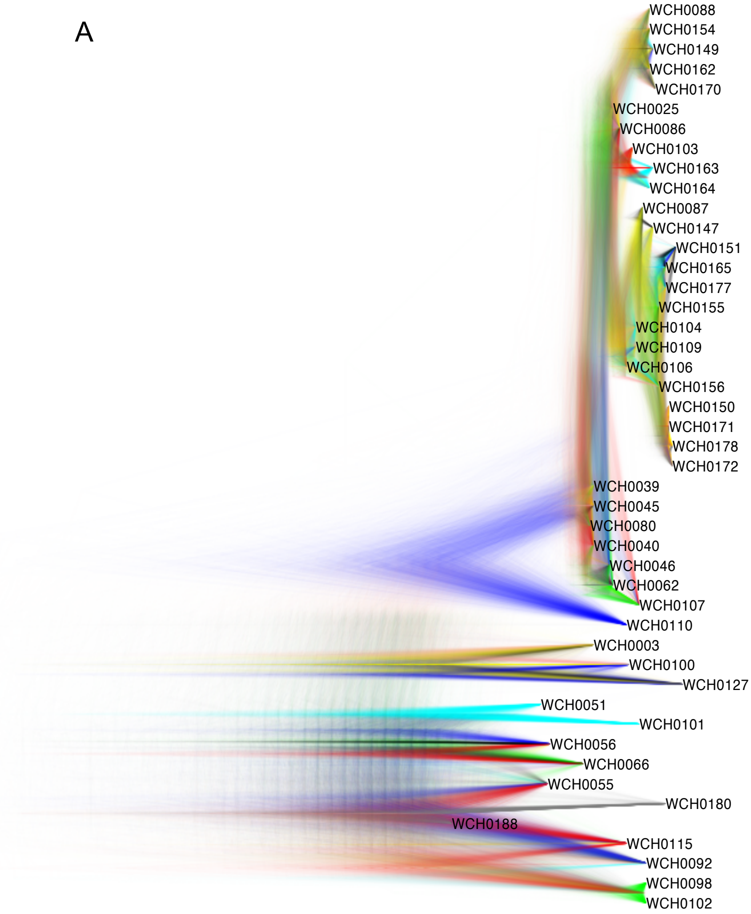
A
WCH0088
WCH0154
WCH0149
WCH0162
WCH0170
WCH0025
WCH0086
WCH0103
WCH0163
WCH0164
WCH0087
WCH0147
WCH0151
WCH0165
WCH0177
WCH0155
WCH0104
WCH0109
WCH0106
WCH0156
WCH0150
WCH0171
WCH0178
WCH0172
WCH0039
WCH0045
WCH0080
WCH0040
WCH0046
WCH0062
WCH0107
WCH0110
WCH0003
WCH0100
WCH0127
WCH0051
WCH0101
WCH0056
WCH0066
WCH0055
WCH0180
WCH0188
WCH0115
WCH0092
WCH0098
WCH0102

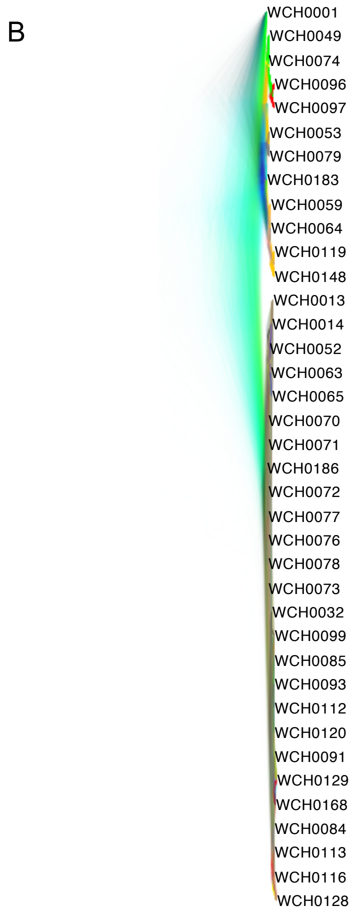
B
WCH0001
WCH0049
WCH0074
WCH0096
WCH0097
WCH0053
WCH0079
WCH0183
WCH0059
WCH0064
WCH0119
WCH0148
WCH0013
WCH0014
WCH0052
WCH0063
WCH0065
WCH0070
WCH0071
WCH0186
WCH0072
WCH0077
WCH0076
WCH0078
WCH0073
WCH0032
WCH0099
WCH0085
WCH0093
WCH0112
WCH0120
WCH0091
WCH0129
WCH0168
WCH0084
WCH0113
WCH0116
WCH0128
